# A biomimetic core-shell nanofibrous dressing for temporally coordinated infection control and mitochondrial protection in diabetic wounds

**DOI:** 10.1016/j.mtbio.2026.103599

**Published:** 2026-08-28

**Authors:** Yanqi Chen, Huihui Zhang, Tingzi Zhao, Hai Zhou, Zhi Xu, Jiaqi Liang, Yixiang HePeng, Chaoyang Huang, Xu Wu, Lianglong Chen, Lei Yang

**Affiliations:** a Department of Burns, Nanfang Hospital, Southern Medical University, Guangzhou, 510515, China; b Department of Thoracic Surgery, Nanfang Hospital, Southern Medical University, Guangzhou, 510515, China; c Huiqiao Medical Center, Nanfang Hospital, Southern Medical University, Guangzhou, 510515, China; d Department of Plastic Surgery, Huizhou Central People's Hospital, No. 41 Eling North Road, Huicheng District, Huizhou City, Guangdong Province, 516001, China

**Keywords:** Core-shell nanofibers, Astragalus polysaccharides, Mitochondrial homeostasis, Mitochondrial metabolic regulation, Diabetic wound healing

## Abstract

Chronic infected diabetic wounds are driven by a self-reinforcing pathological cycle of bacterial infection, oxidative stress, and immune dysregulation, wherein mitochondrial dysfunction contributes to impaired reparative macrophage polarization and angiogenesis. To address this, a skin-mimetic core-shell nanofibrous dressing (AT@PCS) was fabricated via coaxial electrospinning from a pullulan/quaternized chitosan (QCS) matrix, featuring a tannic acid (TA)-crosslinked shell for rapid barrier defense and an Astragalus polysaccharide (APS)-loaded core for sustained intracellular regulation. *In vitro*, the TA/QCS shell achieved >99.6% antibacterial efficiency against *Escherichia coli* and *Staphylococcus aureus* and rapidly scavenged extracellular reactive oxygen species (ROS). Subsequently, sustained APS release supported the activation of Nrf2-mediated antioxidant signaling, improved mitochondrial membrane potential, improved ATP production and mitochondrial respiratory function, and attenuated oxidative stress-induced glycolytic shift, thereby promoting M2 polarization and upregulating VEGF expression. These findings suggest that extracellular ROS scavenging alone is insufficient to fully reverse mitochondrial oxidative damage, underscoring the potential importance of intracellular redox regulation. In a diabetic rat full-thickness infected wound model, AT@PCS achieved 71.7 ± 4.4% wound closure by day 14, significantly superior to the 54.8 ± 3.4% closure rate of the control group, with markedly enhanced neovascularization and orderly collagen remodeling. This temporally coordinated biomimetic platform offers a strategy for coordinated regulation of redox balance, cellular metabolism, and immune microenvironment and provides a potential design strategy for diabetic wound repair.

## Highlights

• Extracellular ROS scavenging alone did not fully alleviate mitochondrial damage.
• APS enhanced Nrf2/HO-1 responses and mitochondrial protection.
• APS-containing fibers favored reparative macrophage remodeling.
• Core-shell fibers coupled early antibacterial defense with sustained APS release.

## 1 Introduction

Chronic infected diabetic wounds represent a severe and refractory complication driven by persistent hyperglycemia and pose substantial threats to patients’ quality of life [1]. A central feature of impaired healing is a self-amplifying pathological cycle in which excessive reactive oxygen species (ROS) accumulation and mitochondrial dysfunction mutually reinforce one another. Within the diabetic microenvironment, persistent hyperglycemia and hyperlipidemia overload cellular energy metabolism, resulting in increased superoxide production from the mitochondrial electron transport chain and triggering oxidative cascades [2]. Excessive ROS in turn disrupt mitochondrial dynamics and exacerbate metabolic dysfunction, perpetuating this positive feedback loop [3]. This mitochondrial impairment compromises oxidative phosphorylation (OXPHOS) in macrophages, promoting a metabolic shift toward glycolysis and favoring a pro-inflammatory macrophage phenotype, thereby limiting anti-inflammatory cytokine production and pro-regenerative growth factor expression [4]. Aberrant ROS accumulation concurrently disrupts the HIF-1α/VEGF axis, impairing angiogenesis and further delaying tissue repair [5]. The nutrient-rich hyperglycemic environment additionally facilitates bacterial adhesion and proliferation, compounding impaired immune defense and ultimately creating a persistent pathological microenvironment [6,7].

In response to these pathological disturbances, existing studies have investigated antibacterial, antioxidative, and immunomodulatory interventions [[8], [9], [10]]. As representative examples, Guo et al. developed a hydrogel-based antioxidant strategy to support mitochondrial function through Nrf2-related signaling [11], whereas Zhou et al. employed a mitochondria-targeted nanoplatform to facilitate macrophage metabolic reprogramming [12]. However, these strategies mainly focus on intracellular redox regulation and do not integrate early infection control with temporally coordinated microenvironment modulation. Improved mitochondrial respiratory function has been linked to macrophage metabolic remodeling, M2 polarization, and the reestablishment of the HIF-1α/VEGF axis [13], a process that is also dependent on effective intracellular ROS clearance as a prerequisite. Extracellular antioxidant capacity alone may therefore be insufficient to fully reverse intracellular mitochondrial oxidative stress. Moreover, emerging evidence indicates that diabetic wounds are characterized by insufficient neutrophil recruitment during the early phase, and indiscriminate anti-inflammatory interventions applied before adequate infection control may paradoxically delay healing [14]. An optimal therapeutic strategy should therefore not uniformly suppress inflammation; rather, it should re-establish the temporal dynamics of the inflammatory response following effective infection control and oxidative stress reduction [3]. How to integrate sustained antibacterial activity, ROS scavenging, mitochondrial protection, and temporally coordinated immune modulation within a single dressing system remains an unresolved and clinically important challenge.

Polysaccharide-based nanofibrous dressings have shown considerable potential for wound repair [15]. Compared with conventional synthetic polymers such as polyvinylpyrrolidone, polyacrylonitrile, and polylactic acid, polysaccharide-derived materials generally offer favorable biocompatibility and biodegradability, which may reduce foreign-body responses and facilitate material clearance after application [16]. Nanofibrous membranes also resemble the fibrous architecture of the native extracellular matrix and provide topographical cues for cell adhesion and migration. Their high surface area and porous structure facilitate the incorporation and sustained release of bioactive agents [17]. In highly exudative wounds, hydrogels may undergo excessive swelling and lose structural stability, while their isotropic structure provides limited directional guidance for cell migration [18]. Nanofibrous membranes may offer better structural support and fluid transport while enabling sustained delivery of antimicrobial agents [19]. In this study, pullulan and quaternized chitosan (QCS) were selected to construct the fibrous matrix. Pullulan provides water solubility, film-forming ability, and biodegradability [20], whereas QCS provides antibacterial activity through its permanently charged quaternary ammonium groups [21]. The positively charged groups of QCS may interact electrostatically with negatively charged bacterial surfaces and disrupt membrane integrity, resulting in leakage of intracellular components [22].

Two bioactive components were incorporated with the aim of supporting mitochondrial function and modulating the metabolic and immune microenvironment. Astragalus polysaccharide (APS), a principal bioactive polysaccharide of *Astragalus membranaceus* [23], has been reported to exhibit antioxidant, immunomodulatory, and pro-angiogenic activities associated with Nrf2 signaling [24] and with macrophage phenotypic regulation [25]. These properties make APS an attractive candidate for incorporation into the core layer for sustained regulation of the oxidative and immune microenvironment. Tannic acid (TA) is a natural polyphenol rich in phenolic hydroxyl groups that enable physical crosslinking with polysaccharide chains through hydrogen bonding and other noncovalent interactions [26], thereby avoiding the use of conventional chemical crosslinkers such as glutaraldehyde. These phenolic groups also contribute to interfacial adhesion and ROS scavenging [27], while interactions with bacterial surfaces and biofilm matrix components may confer antibacterial activity and inhibition of biofilm formation [28].

**Scheme 1:**
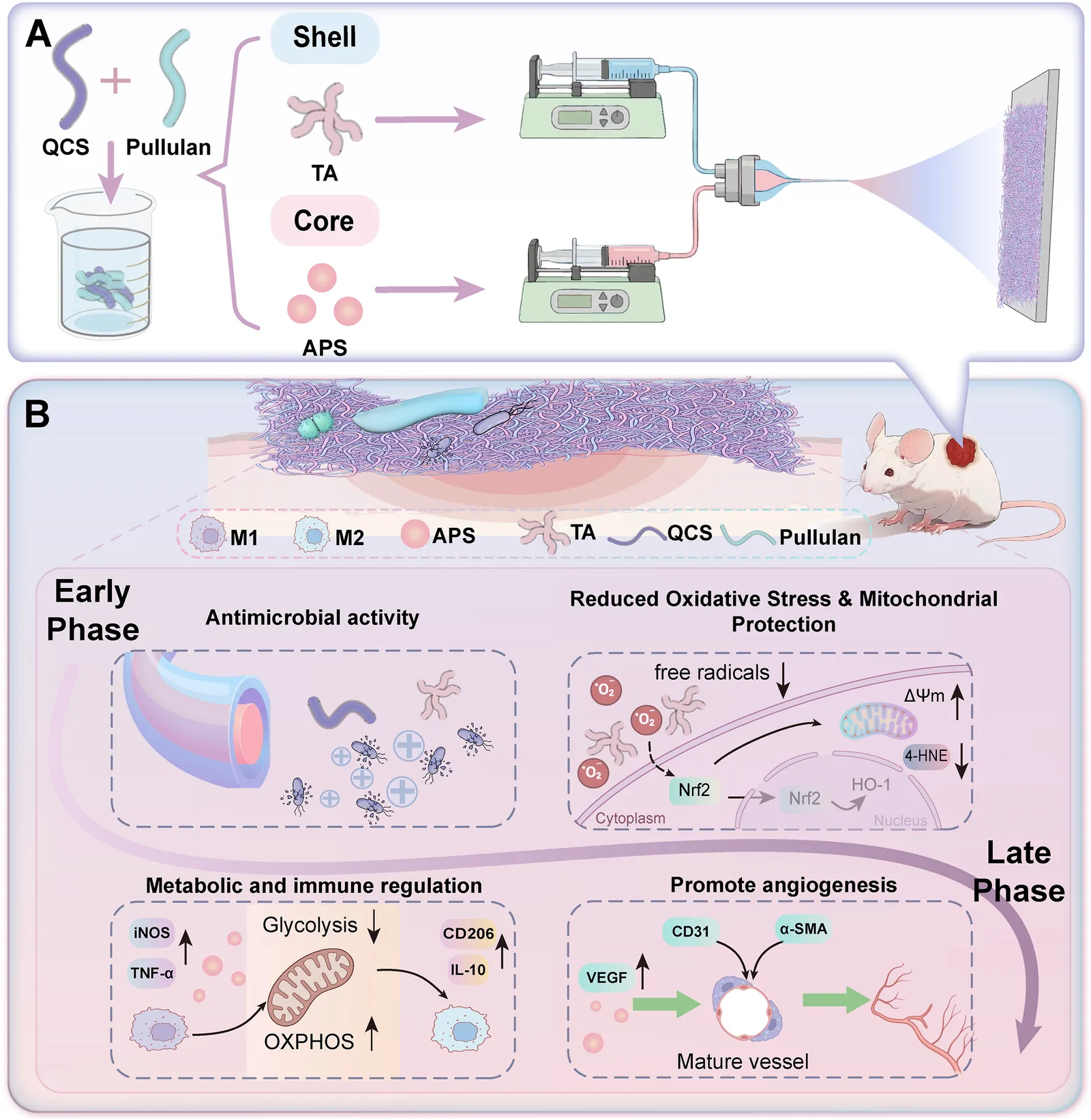
**Preparation and application of AT@PCS core-shell polysaccharide nanofibrous dressings in the repair of infected diabetic wounds. (A)** Schematic illustration of the fabrication of AT@PCS core-shell nanofibrous dressings. **(B)** AT@PCS dressings exhibited a functionally staged release profile, providing early antibacterial activity and ROS scavenging to modulate the oxidative microenvironment, together with sustained APS delivery associated with mitochondrial bioenergetic recovery and M2 macrophage polarization, thereby supporting angiogenesis and tissue regeneration in diabetic wounds.

Native skin integrates barrier defense, immune regulation, and tissue repair through the multilayered organization of the stratum corneum, viable epidermis, and dermis [29]. Many polysaccharide-based electrospun dressings remain single-component monolayer membranes or simply blended membranes, whereas existing core-shell systems primarily focus on compartmentalized loading and release control [30]. Although QCS, TA, and APS have been incorporated into wound-repair systems for antibacterial, antioxidant, or pro-reparative purposes [31,32], their spatial and temporal organization within a polysaccharide-based fibrous platform remains less explored. Inspired by the hierarchical organization of native skin, we fabricated a core-shell nanofibrous membrane, denoted AT@PCS, using coaxial electrospinning. The dressing consists of a pullulan/QCS fibrous matrix with a TA-crosslinked shell and an APS-loaded core. The shell was designed to provide immediate contact-active antibacterial and extracellular ROS-scavenging effects, whereas the core was designed to enable sustained APS release for subsequent regulation of the oxidative and immune microenvironment. The key design feature of AT@PCS lies not in the individual use of a core-shell structure, TA/QCS, or APS, but in their spatiotemporal organization within a single polysaccharide-based fibrous platform. The resulting dressing was evaluated in terms of its physicochemical properties, release behavior, antibacterial and antioxidant activity, mitochondrial bioenergetics, immunomodulatory effects, and therapeutic efficacy in infected diabetic wounds (Scheme 1).

## 2 Materials and methods

### 2.1 Materials

Pullulan, quaternized chitosan, astragalus polysaccharide, and tannic acid were purchased from Macklin Biochemical Co., Ltd. (Shanghai, China). Glacial acetic acid was obtained from Sinopharm Chemical Reagent Co., Ltd. (Shanghai, China). The Calcein-AM/PI cell live/dead staining kit and DMAO/PI bacterial live/dead staining kit were purchased from UElandy and Beyotime Biotechnology Co., Ltd., respectively. Phalloidin (iFluor 488, CA1610) was obtained from Zeye Biotechnology (Hangzhou, China), and 4′,6-diamidino-2-phenylindole (DAPI, ID2250) was obtained from Solarbio (Beijing, China). Crystal violet (CA0059) was purchased from Maige Biotechnology Co., Ltd. Human umbilical vein endothelial cells (HUVECs) were procured from Bioher Biotechnology Co., Ltd. (Shanghai, China). L929 fibroblasts and RAW 264.7 macrophages were supplied by the Clinical and Medical Testing Center, Nanfang Hospital, Southern Medical University. Unless otherwise specified, all other chemical reagents were of analytical grade, purchased from Solarbio Technology Co., Ltd. (Beijing, China), and were used without further purification.

### 2.2 Preparation of electrospun nanofibrous membranes

A blended polysaccharide solution composed of pullulan and QCS was used as the base spinning solution. According to preliminary optimization, the concentrations of pullulan and QCS were set at 16% and 2%, respectively. Briefly, pullulan was dissolved in distilled water with overnight stirring at 25 °C until completely dissolved. QCS was then added to the pullulan solution containing 1% glacial acetic acid and further stirred for 4 h to obtain a homogeneous spinning solution, designated as PCS.

To prepare the core spinning solution, APS was added to the PCS solution at a concentration of 4% and stirred at room temperature until completely dissolved. The resulting solution was designated as A@PCS. For the shell spinning solution, TA was dissolved in the PCS solution at a concentration of 7.5%, and the obtained solution was designated as T@PCS.

Core-shell nanofibrous membranes were fabricated by coaxial electrospinning, with A@PCS and T@PCS used as the core and shell solutions, respectively. The two solutions were delivered through the inner and outer channels of a coaxial needle. Based on preliminary optimization, the flow rates of the core and shell solutions were set at 0.015 and 0.03 mL/min, respectively. The applied voltage was 24.6 kV, the tip-to-collector distance was 15 cm, and the ambient temperature was maintained at 28 °C. Aluminum foil was used as the grounded collector, and electrospinning continued for 4 h. After spinning, the obtained membranes were dried at 50 °C for 2 h and stored in a desiccator at room temperature until further use.

### 2.3 Physicochemical characterization of materials

The surface microstructure of the electrospun nanofibrous membranes was characterized using scanning electron microscopy (SEM). Fiber diameters were measured using ImageJ software (National Institutes of Health), with at least 100 fibers analyzed per group. Fourier transform infrared spectroscopy (FTIR) was employed to characterize the characteristic functional groups of the polysaccharide components as well as the overall chemical structure of the nanofibrous membranes.

The surface wettability of the membranes was evaluated using a contact angle analyzer. A 5 μL droplet of deionized water was placed on the sample surface, and the dynamic spreading process until complete disappearance of the droplet was recorded in real time. Images were extracted frame by frame from the recorded video to calculate the contact angle, thereby quantitatively evaluating the hydrophilic or hydrophobic properties of the membrane surface.

The temporal release of APS was evaluated by a drug release assay. Approximately 100 mg of each A@PCS or AT@PCS nanofibrous membrane sample was accurately weighed and immersed in phosphate-buffered saline (PBS, pH 5.4 or 7.4) to evaluate APS release under acidic and near-physiological pH conditions. The release study was conducted at 37 °C under constant shaking at 100 rpm. The release behavior of APS was investigated using the dialysis method. The nanofibrous membranes were dispersed in 31.25 mL of buffer solution and transferred into a pretreated cellulose dialysis bag, which was then sealed and immersed in 50 mL of the corresponding buffer solution. At predetermined time points, 1.0 mL of the release medium was withdrawn and immediately replaced with an equal volume of fresh buffer prewarmed to 37 °C. The concentration of released APS was quantified by high-performance liquid chromatography coupled with evaporative light-scattering detection (HPLC-ELSD), with the detailed chromatographic conditions provided in the Supplementary Methods. The cumulative release percentage was calculated using the following equation: Cumulative release (%) = (M_t_/M_L_) × 100, where M_t_ represents the cumulative amount of APS released at time t, and M_L_ denotes the total APS loading in the nanofibrous membrane sample.

The adhesiveness and flexibility of AT@PCS were evaluated. Samples (1 cm × 4 cm) were applied to the finger joints of volunteers, and a small amount of distilled water was used to moisten the interface to ensure adequate adhesion. The fingers were then bent sequentially to 90°, 120°, and 180°, with each position maintained for 30 s to simulate dynamic deformation during joint movement. At each bending angle, the samples were examined for failure phenomena such as edge lifting, detachment, or interlayer delamination, and the adhesion state at the interface was recorded in real time using a digital camera.

### 2.4 Antibacterial evaluation of materials

The antibacterial activity of the nanofibrous membranes was systematically evaluated using plate colony counting, bacterial live/dead fluorescence staining, and SEM morphological analysis, together with crystal violet staining to assess their ability to inhibit biofilm formation. The membranes were cut into circular discs with a diameter of 8 mm and co-incubated with 2 mL of bacterial suspension containing *Escherichia coli* (*E. coli*) or *Staphylococcus aureus* (*S. aureus*) (1 × 10^6^ CFU/mL) at 37 °C under constant agitation for 24 h. After incubation, the bacterial suspensions were serially diluted, and 100 μL aliquots from each dilution were evenly spread onto LB agar plates. The plates were further incubated at 37 °C for 24 h, followed by enumeration of colony-forming units (CFU). Bacterial suspensions without membrane treatment served as the blank control. The antibacterial rate was calculated as follows: (N_control_ - N_sample_)/N_control_ × 100%.

Bacterial live/dead staining was performed to evaluate antibacterial efficacy. Briefly, 1 mL of the co-cultured bacterial suspension was centrifuged at 12,000 rpm for 5 min, and the supernatant was discarded. The pellet was washed twice with PBS and then resuspended in 1 mL of PBS. Fluorescent staining was conducted using a DMAO/PI Live/Dead Bacterial Staining Kit (Beyotime, C2030S) according to the manufacturer's instructions, followed by incubation in the dark for 15 min. Subsequently, 10 μL of the bacterial suspension was pipetted onto a glass slide, and fluorescence images were acquired using an inverted fluorescence microscope using the green channel (live bacteria) and the red channel (dead bacteria). The relative antibacterial rates of two bacterial species were quantified.

SEM was employed to observe morphological changes in bacteria after antibacterial treatment. Bacteria not exposed to the nanofibrous membranes were used as the control group, while those co-incubated with the membranes served as the experimental group. Samples were fixed with 2.5% glutaraldehyde for 2 h, followed by sequential dehydration in graded ethanol solutions (30%–100%). The samples were then vacuum-dried and sputter-coated with gold. Finally, the ultrastructural changes of the bacteria were examined using SEM.

Crystal violet staining was used to evaluate the effect of the materials on bacterial biofilm formation. The nanofibrous membrane samples were placed at the bottom of 24-well plates, and 500 μL of *E. coli* or *S. aureus* suspension (approximately 10^6^ CFU/mL) was added to each well. The plates were incubated statically at 37 °C for 72 h to induce biofilm formation. After incubation, the samples were gently washed three times with PBS to remove non-adherent planktonic bacteria, followed by staining with 0.1% crystal violet solution at room temperature for 20 min. The samples were then thoroughly rinsed with deionized water to remove excess dye. After drying, the crystal violet-stained biofilm biomass was observed and photographed.

### 2.5 Biocompatibility assays

Either extracts of the nanofibrous membranes or direct co-culture with the membranes was used for the cell experiments. The hemolysis assay, CCK-8 assay, live/dead cell staining, EdU proliferation, and Transwell migration assays were sequentially performed to evaluate the biocompatibility of the materials. The hemolysis assay was conducted to assess blood compatibility. Fresh anticoagulated porcine whole blood was collected, centrifuged and washed, and the resulting erythrocytes were subsequently diluted with sterile normal saline (0.9% NaCl) to obtain a 2% (v/v) red blood cell suspension. The pretreated nanofibrous membrane samples were co-incubated with 1 mL of the red blood cell suspension at 37 °C for 1 h. Normal saline (0.9%) and Triton X-100 were used as the negative and positive controls, respectively. After incubation, the samples were centrifuged at 5000 rpm for 10 min, and the supernatant was collected and transferred to a 96-well plate. The absorbance (OD) was measured at 540 nm. The hemolysis rate was calculated as follows: Hemolysis (%) = [(OD_sample_ − OD_negative control_)/(OD_positive control_ - OD_negative control_)] × 100% (n = 3).

The *in vitro* cytocompatibility of the materials was evaluated using the CCK-8 assay. L929 cells were seeded in 96-well plates at a density of 1 × 10^4^ cells per well and cultured for 12 h to ensure adequate attachment. The original culture medium was then discarded, and the corresponding nanofibrous membrane extracts were added directly to the cells for the designated incubation period. After treatment, an appropriate volume of CCK-8 working solution was added to each well and incubated in the dark for 2 h. The absorbance was measured at 450 nm using a microplate reader. Untreated cells served as the control group, and cell viability was assessed by comparing the OD values among groups.

Live/dead cell staining was further performed to evaluate the cytocompatibility of the materials. HUVECs were seeded in 96-well plates at a density of approximately 1 × 10^4^ cells per well and cultured for 12 h to allow sufficient adhesion. The medium was then replaced with nanofibrous membrane extracts for treatment. Cells cultured in standard medium served as the control. At the designated time points, the cells were gently washed with PBS, followed by incubation with the calcein-AM/PI staining working solution in the dark for 15 min. After staining, fluorescence images were obtained using an inverted fluorescence microscope, with live cells emitting green fluorescence and dead cells emitting red fluorescence. Quantitative analysis of fluorescence signals was performed using ImageJ software to assess cell viability and cytotoxicity.

The EdU incorporation assay was performed to evaluate the effect of the nanofibrous membranes on cell proliferation. At predetermined time points, EdU working solution was added and incubated with the cells to label those in the DNA synthesis phase. The cells were then fixed and permeabilized, followed by fluorescent labeling via click chemistry according to the manufacturer's instructions. Cell nuclei were counterstained with DAPI. Fluorescence images were acquired using an inverted fluorescence microscope. The numbers of EdU-positive cells and total cells were quantified, and the EdU-positive rate was calculated to assess cell proliferative activity and DNA synthesis activity.

A Transwell migration assay was performed to evaluate the effect of the nanofibrous membranes on cell migratory capacity. L929 cells were serum-starved in serum-free medium for 12 h, then trypsinized and resuspended in serum-free medium. Cells were seeded into the upper chamber of the Transwell inserts at a density of approximately 1 × 10^4^ cells per well in 200 μL of serum-free medium. The lower chamber was filled with culture medium containing nanofibrous membrane extracts, whereas standard medium was used for the control group. The cells were incubated at 37 °C in a 5% CO_2_ atmosphere for a specified period. After incubation, non-migrated cells on the upper surface of the membrane were gently removed with a cotton swab. Cells that had migrated to the lower surface were fixed with 4% paraformaldehyde for 20 min and stained with 0.1% crystal violet for 15 min. Images were then captured under a light microscope, and migrated cells were counted and statistically analyzed.

### 2.6 Antioxidant assays

Antioxidant activity was assessed using DPPH and ABTS radical scavenging assays by measuring absorbance at 517 nm and 734 nm, respectively. Vitamin C (VC) was used as the positive control, whereas the corresponding radical working solution without a material sample served as the control. In the DPPH assay, samples from each group were mixed with an ethanolic DPPH solution and incubated in the dark. The supernatant was subsequently collected, and the absorbance was measured at 517 nm. The DPPH radical scavenging rate was calculated based on the control absorbance as follows: DPPH scavenging (%) = (A_c_ − A_H_)/A_c_ × 100%, where A_c_ is the absorbance of the control and A_H_ is the absorbance of the sample. In the ABTS assay, the ABTS working solution was prepared, diluted, and subsequently mixed with the samples at a 1:1 vol ratio. After incubation in the dark at room temperature, the absorbance was measured at 734 nm, and the radical scavenging capacity toward ABTS radicals was calculated from the change in absorbance.

Hydrogen peroxide (H_2_O_2)_ and superoxide anion (O_2_^·-^) scavenging assays were also performed. Equal masses of nanofibrous membranes from each group were used for the assays. For H_2_O_2_ scavenging, the membranes were incubated with an H_2_O_2_ solution, and the residual H_2_O_2_ content was determined using a commercial assay kit (BC3595, Solarbio, Beijing, China) according to the manufacturer's instructions, with absorbance measured at 415 nm. O_2_^·-^ scavenging activity was evaluated using the NADH-PMS-NBT system. After incubation with the membrane samples for 5 min, NBT reduction was measured at 560 nm. The scavenging efficiency was calculated as (A_0_ − A_s_)/A_0_ × 100%, where A_0_ and A_s_ represent the absorbance values of the control and sample-treated systems, respectively, after subtraction of the corresponding background absorbance. All measurements were performed in triplicate.

Intracellular ROS levels were detected using a DCFH-DA fluorescent probe. Briefly, HUVECs were divided into a normal control group, a high-glucose/H_2_O_2_ (HG/H_2_O_2_) model group, and material-treated groups. The HG/H_2_O_2_ model was established by culturing cells in medium containing 25 mM D-glucose and 100 μM H_2_O_2_. The material-treated groups were incubated with extracts derived from PCS, A@PCS, T@PCS, or AT@PCS under the same HG/H_2_O_2_ stimulation. After treatment, the cells were incubated with DCFH-DA working solution at 37 °C in the dark for 30 min, washed with PBS, and observed using an inverted fluorescence microscope. Intracellular ROS levels were quantified from the green fluorescence intensity using ImageJ.

To further evaluate oxidative damage and the endogenous antioxidant response, cells were subjected to the same HG/H_2_O_2_ stimulation. The HG/H_2_O_2_ model group received the HG/H_2_O_2_ stimulation without material extracts, whereas the treatment groups were incubated with extracts derived from PCS, A@PCS, T@PCS, or AT@PCS. Immunofluorescence staining was performed to evaluate 4-HNE accumulation and Nrf2. After blocking, the RAW 264.7 cells were incubated with primary antibodies at 4 °C overnight, followed by incubation with fluorescence-labeled secondary antibodies and nuclear counterstaining with DAPI. Images were acquired using an inverted fluorescence microscope, and the fluorescence signals of 4-HNE and Nrf2 were quantitatively analyzed using ImageJ. Mitochondrial membrane potential (MMP) was assessed using JC-1 staining in HUVECs and RAW 264.7 cells. JC-1 powder was dissolved according to the manufacturer's instructions in DMSO to prepare a 2 mg/mL stock solution, which was then diluted to a working concentration of 2 μM. The JC-1 working solution was added to the cell cultures and incubated at 37 °C in the dark for 30 min, followed by three gentle washes with PBS to remove excess dye. Fluorescence images were subsequently obtained using a confocal laser scanning microscope. Cells with a high membrane potential exhibited red fluorescence (JC-1 aggregates), whereas those with low membrane potential showed green fluorescence (JC-1 monomers).

A scratch migration assay was performed to evaluate the effect of nanofibrous membranes on cell migration under oxidative stress conditions. L929 cells were seeded in 6-well plates and cultured until a fully confluent monolayer was formed. A straight scratch was created across the cell layer using a sterile 200 μL pipette tip held perpendicular to the plate. The cells were then gently washed to remove detached cells and cultured in low-serum medium containing 1% FBS. For the treatment groups, nanofibrous membrane extracts were added to medium containing 25 mM D-glucose and 100 μM H_2_O_2_, whereas the control group received the same HG/H_2_O_2_ stimulation without material extracts. Images of the scratch area were captured at 0, 36, and 72 h using an inverted microscope. The wound area was quantitatively analyzed using ImageJ software, and the scratch closure rate was calculated.

Western blotting was performed to assess Nrf2 and HO-1 protein expression. Cells were subjected to the indicated interventions for the specified durations and then lysed for protein extraction. Equal amounts of protein were separated by SDS-PAGE and electrotransferred to polyvinylidene difluoride membranes. The membranes were blocked with 5% (w/v) BSA in TBST (50 mM Tris-HCl, 150 mM NaCl, and 0.05% Tween 20; pH 7.4) for 1 h at room temperature and then incubated with the indicated primary antibodies overnight at 4 °C. After three 10-min washes with TBST under gentle agitation, the membranes were incubated with species-matched HRP-conjugated secondary antibodies (1:5000) for 1 h at room temperature. Following additional TBST washes, protein bands were visualized using enhanced chemiluminescence.

### 2.7 Immunomodulation assays

RAW 264.7 macrophages were used to evaluate the immunomodulatory effects of the nanofibrous membranes. Cells in the control group were maintained in normal culture medium, whereas those in the HG/LPS and material-treated groups were treated with medium containing 25 mM D-glucose and 200 ng/mL LPS for 24 h to establish an inflammatory macrophage model. The medium in all groups was then replaced. The control and HG/LPS groups were cultured in fresh medium without material extracts, whereas the material-treatment groups were incubated with the corresponding nanofibrous membrane extracts. After treatment, immunofluorescence staining was performed to assess the expression of TNF-α, IL-6, IL-10, iNOS, CD163, and CD206. Cells were fixed with 4% paraformaldehyde and permeabilized with 0.1% Triton X-100, followed by overnight incubation with the corresponding primary antibodies at 4 °C. Subsequently, fluorescence-labeled secondary antibodies were added for 1 h at room temperature in the dark, and nuclei were counterstained with DAPI. Fluorescence images were acquired using a confocal laser scanning microscope (ZEISS, LSM 980), and fluorescence intensity was quantitatively analyzed using ImageJ software to assess macrophage phenotype-associated changes induced by the nanofibrous membrane extracts.

For flow cytometric analysis of macrophage phenotypes, cells were seeded in 6-well plates and treated according to the experimental design. After treatment, cells were collected, centrifuged, and resuspended in PBS. Cells were first incubated with an anti-CD86 antibody in the dark, followed by washing, fixation, and permeabilization with Triton X-100. The cells were then incubated with an anti-CD206 antibody in the dark. Following staining, cells were washed, resuspended in PBS, and analyzed using a flow cytometer. CD86^+^CD206^-^ and CD86^−^CD206^+^ cells were defined as M1-associated and M2-associated macrophage populations, respectively, and their proportions were quantified using FlowJo software.

### 2.8 Angiogenesis assays

HUVECs were used to evaluate the pro-angiogenic activity of the nanofibrous membrane extracts. Immunofluorescence staining was performed to evaluate VEGF expression. HUVECs were seeded in 24-well plates and cultured until adherent, followed by HG/LPS stimulation. The medium was then replaced. The HG/LPS group was cultured in fresh medium without material extracts, whereas the material-treatment groups were cultured with the corresponding nanofibrous membrane extracts for 24 h. After treatment, the cells were fixed with 4% paraformaldehyde, permeabilized, and blocked, and then incubated overnight at 4 °C with an anti-VEGF primary antibody. Subsequently, the cells were incubated with the corresponding fluorescence-labeled secondary antibody for 1 h in the dark, and the nuclei were counterstained with DAPI. Fluorescence images were acquired using a confocal laser scanning microscope. All groups were imaged under identical acquisition settings, and VEGF fluorescence intensity was quantitatively analyzed using ImageJ software.

For the tube formation assay, Matrigel was added to 24-well plates and allowed to polymerize completely. Following HG/LPS stimulation, HUVECs were collected and seeded onto the polymerized Matrigel at a density of 3.0 × 10^4^ cells per well. The HG/LPS group was cultured in fresh medium without material extracts, whereas the material-treatment groups were cultured with the corresponding nanofibrous membrane extracts for 12 h in a humidified incubator at 37 °C in 5% CO_2_. After incubation, the cells were stained with a calcein-AM/PI live/dead cell staining kit, and the formation of capillary-like networks was observed under a fluorescence microscope. For each sample, three randomly selected fields were imaged and averaged before statistical analysis. The number of junctions was quantified using ImageJ software to assess tube formation.

### 2.9 *In vivo* evaluation of diabetic wound healing

All animal experimental procedures were approved by the Laboratory Animal Welfare and Ethics Committee of Nanfang Hospital, Southern Medical University (Approval No. IACUC-LAC-20251024-005), and were conducted in compliance with the Guide for the Care and Use of Laboratory Animals and relevant national ethical guidelines. Male Sprague-Dawley (SD) rats (10 weeks old, 200 ± 10 g) were purchased from Zhuhai Bestest Biotechnology Co., Ltd. and housed in an SPF-certified facility under controlled conditions (12 h light/dark cycle, 22 ± 2 °C, and 50 ± 10% relative humidity) with food and water available ad libitum. All animals underwent a 7-day acclimatization period prior to any experimental manipulation.

A streptozotocin (STZ)-induced type 1 diabetic rat model of infected full-thickness wounds was established to evaluate the wound healing efficacy of nanofibrous membranes. Sixty rats were randomly assigned to five groups: control, PCS, A@PCS, T@PCS, and AT@PCS, with 12 rats per group. Type 1 diabetes was induced by a single intraperitoneal injection of STZ (50 mg/kg), freshly dissolved in 0.1 M sodium citrate buffer (pH 4.5). Rats with random blood glucose levels higher than 16.7 mmol/L, measured 72 h after STZ injection, were considered diabetic. Seven days after STZ injection, following intraperitoneal anesthesia with 2% sodium pentobarbital (50 mg/kg), the dorsal hair was removed, and four full-thickness excisional wounds (each 10 mm in diameter) were created on the dorsal skin of each rat using a sterile biopsy punch. For wound closure analysis, the four wounds from each rat were first averaged at each time point, and this animal-level mean was used as one independent biological replicate for statistical analysis (n = 3 animals per group at each time point).

To establish the infected wound model, immediately after wound creation (day −2), each wound bed was evenly inoculated with 20 μL of an *S. aureus* suspension (5 × 10^7^ CFU/mL). The infection was allowed to establish for 48 h, after which the end of this period was designated as treatment day 0, and the respective dressings were applied. Sterile gauze was applied to the control wounds. All wounds within the same animal received the same treatment. On days 3, 7, 14, and 21, three rats from each group were humanely euthanized for tissue sample harvesting. Dressings were changed every 72 h, and wound healing was documented by digital photography. Wound areas were quantified using ImageJ software, and the wound closure rate was calculated as follows: Wound closure rate (%) = (S_0_ − S_t_)/S_0_ × 100, where S_0_ is the initial wound area and S_t_ is the wound area at the indicated time point.

For *in vivo* antibacterial assessment, wound exudates were collected on day 3 using sterile cotton swabs, eluted into 1 mL of sterile PBS, and serially diluted. Aliquots (100 μL) were spread onto LB agar plates and incubated at 37 °C for 24 h. The number of bacterial colonies was counted to evaluate the *in vivo* antibacterial efficacy of the different dressings.

### 2.10 Histological analysis

Collected tissue samples were fixed in 4% paraformaldehyde, embedded in paraffin, and cut into sections. Hematoxylin and eosin (H&E) staining was performed on wound tissues collected on treatment days 3 and 7; day 7 tissues were additionally used for Masson's trichrome, immunohistochemical, and immunofluorescence staining, whereas day 21 tissues were used for Sirius Red staining. H&E staining was performed to observe histomorphological changes. Masson's trichrome staining was used to evaluate collagen deposition and arrangement, while Sirius Red-stained sections were examined under polarized light microscopy to assess collagen fiber composition and remodeling. Immunohistochemical staining for IL-10, TNF-α, and Nrf2 was conducted to assess inflammatory responses and the endogenous antioxidant response during wound healing. Immunofluorescence staining for iNOS, CD206, α-SMA, and CD31 was performed to analyze macrophage phenotype-associated marker expression, angiogenesis, and vascular maturation. All histological and immunostaining images were quantitatively analyzed using ImageJ software. For histological and immunostaining analyses, measurements from multiple tissue sections or microscopic fields obtained from the same rat were first averaged, and the resulting animal-level value was used as one independent biological replicate for statistical analysis.

### 2.11 Statistical analysis

All quantitative data were statistically analyzed and visualized using Origin 2024 and GraphPad Prism 10.1. Data are presented as the mean ± standard deviation (SD). Unless otherwise stated, experiments were performed with at least three independent biological replicates. Technical replicates, including repeated measurements and multiple microscopic fields or tissue sections from the same sample, were averaged before statistical analysis and were not treated as independent biological replicates. For *in vivo* wound closure, histological, and immunofluorescence analyses, the animal was used as the statistical unit. Data normality was assessed using the Shapiro-Wilk test. Comparisons between two groups were performed using an unpaired two-tailed Student's t-test, whereas comparisons among multiple groups were performed using one-way analysis of variance (one-way ANOVA) followed by Tukey's multiple comparisons test. Wound closure over time was analyzed using two-way ANOVA followed by Tukey's post hoc test. Statistical significance was denoted as “ns,” not significant; *p < 0.05, **p < 0.01, ***p < 0.001, and ****p < 0.0001.

## 3 Results

### 3.1 Fabrication and characterization of core-shell nanofibrous dressings

The hierarchical architecture of native skin confers a distinctive temporal functional logic, wherein the epidermal layer establishes dual physicochemical barriers through keratinocytes and antimicrobial peptides, while the dermal layer maintains tissue homeostasis and regenerative capacity via immunomodulation and vascular remodeling [14]. Inspired by this outside-in defense-regeneration architecture, the AT@PCS core-shell nanofibrous membranes were fabricated via coaxial electrospinning, employing a pullulan/QCS composite solution. The hydroxyl groups on pullulan chains interact with the quaternary ammonium groups and amino groups of QCS through hydrogen bonding and electrostatic interactions, promoting intermolecular physical entanglement and enhancing the viscoelasticity of the blended solution, thereby enabling pullulan-facilitated electrospinning of QCS [33]. Coaxial electrospinning was employed to encapsulate APS within the fiber core while incorporating TA as a physical crosslinker in the shell layer, where the shell provides interfacial protection and the core enables sustained drug release, thereby mimicking the functional stratification of native skin.

**Fig. 1:**
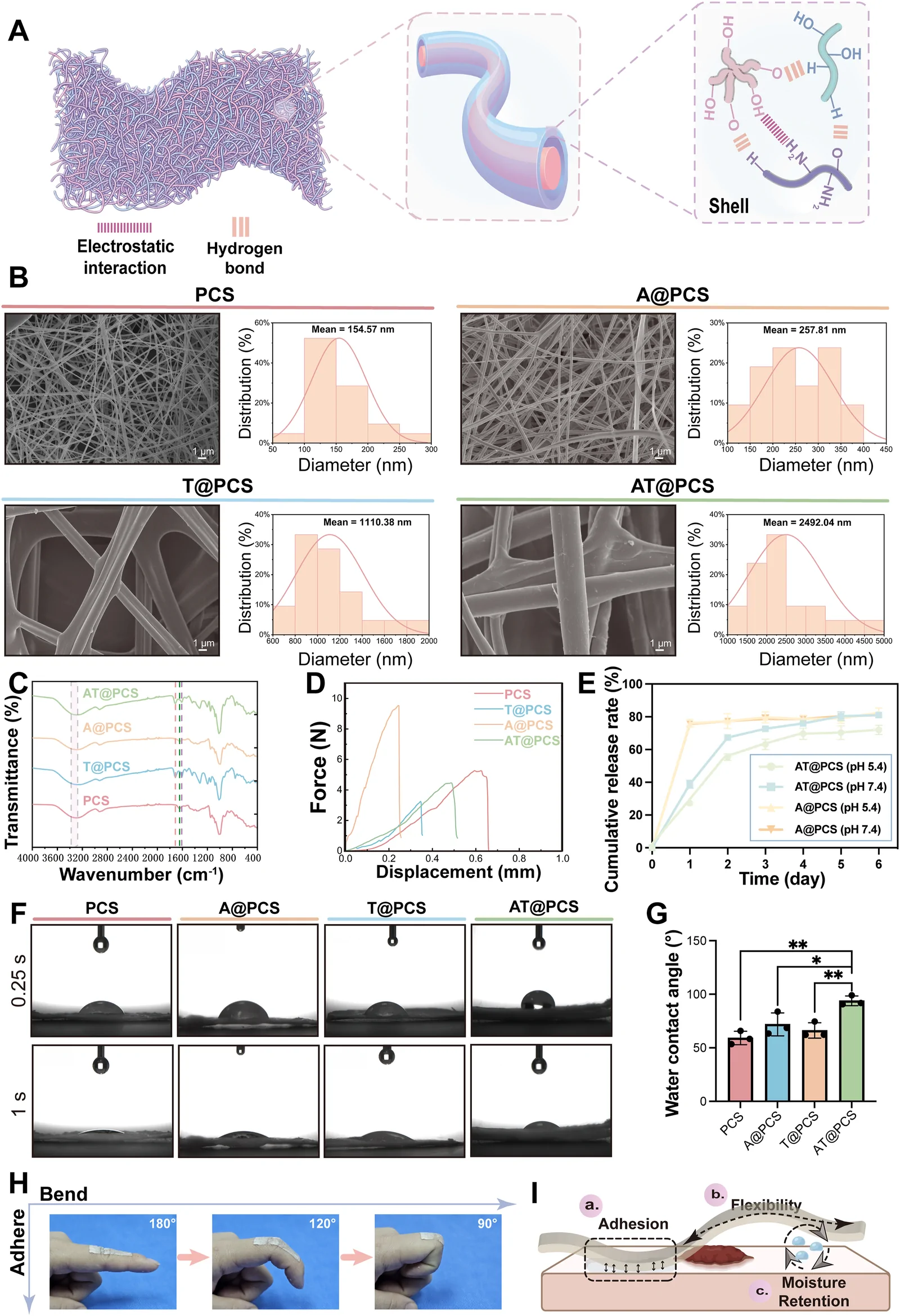
**Design and construction of a core-shell polysaccharide nanofiber dressing with a multifunctional interface. (A)** Schematic illustration of the core-shell polysaccharide nanofiber dressing architecture, wherein pullulan, QCS, and TA are assembled into a stable three-dimensional network through electrostatic interactions and hydrogen bonding. **(B)** SEM images of PCS, A@PCS, T@PCS, and AT@PCS nanofibers and their corresponding nanofiber diameter distributions. **(C)** FTIR spectra of nanofiber membranes across all experimental groups. **(D)** Force-displacement curves of nanofiber membranes across all experimental groups. **(E)***In vitro* cumulative release profiles of APS from the AT@PCS and A@PCS systems at pH 5.4 and pH 7.4 (37 °C). **(F)** Droplet spreading images of nanofiber membranes across all groups captured at 0.25 s and 1 s. **(G)** Corresponding quantitative analysis of WCAs. **(H)** Characterization of the adhesion and flexibility of AT@PCS nanofiber membranes at varying finger flexion angles (180°, 120°, and 90°). **(I)** Schematic illustration of the functional performance of AT@PCS nanofiber membranes in wound applications, highlighting their adhesive properties, mechanical flexibility, and moisture retention capacity.

Given the intrinsic hydrophilicity of the polysaccharide matrix, wet-state stability represents a critical limiting factor for the application of nanofibrous dressings in dynamic wound environments. PCS and A@PCS underwent dissolution or swelling into a hydrogel state under wet conditions, resulting in a severe loss of structural integrity and consequent inability to maintain effective wound coverage and interfacial stability. In contrast, T@PCS and AT@PCS, which were modified through TA-mediated physical crosslinking, retained structural integrity and withstood mechanical handling after thorough wetting (Fig. S1A and B). As illustrated in Fig. 1A, the polyphenolic hydroxyl groups of TA form a dense three-dimensional crosslinked network with polysaccharide chain segments through electrostatic interactions and hydrogen bonding, which enhances the resistance of the fibrous membrane to external fluid penetration and maintains interfacial structural integrity, thereby overcoming the inherent limitations of polysaccharide-based nanofibrous membranes in moist wound environments. SEM characterization further revealed that all four groups of fibers exhibited continuous and uniform filamentous morphology without bead formation or fiber breakage defects, confirming that neither TA crosslinking nor APS loading disrupted the continuity of the electrospinning process (Fig. 1B). The average diameters of PCS, A@PCS, T@PCS, and AT@PCS fibers were 154.57 ± 42.79 nm, 257.81 ± 79.52 nm, 1110.38 ± 301.55 nm, and 2492.04 ± 938.02 nm, respectively, showing an overall increase following APS and/or TA incorporation and the construction of the coaxial core-shell structure. This increase may be associated with the increased solid content and enhanced intermolecular interactions following APS and TA incorporation. In addition, the combined core and shell flow streams during coaxial electrospinning may generate a larger compound jet than conventional single-fluid electrospinning, further contributing to the increased fiber diameter. The mean diameter of AT@PCS fibers was approximately 2.49 μm, approaching the micrometer scale of native dermal collagen fiber bundles, thereby providing some morphological similarity to the fibrous extracellular matrix [34]. The increased fiber dimensions may also influence interfiber spacing, fluid-handling behavior, and the diffusion path of APS from the core layer.

FTIR spectra confirmed the successful integration of pullulan, QCS, TA, and APS within the nanofibers (Fig. 1C). All four sample groups exhibited a broad and intense overlapping stretching vibration peak attributed to O-H and N-H at approximately 3280 cm^−1^, alongside a saturated C-H stretching vibration peak at approximately 2900 cm^−1^, collectively confirming the presence of the polysaccharide backbone. Relative to PCS, the TA-containing groups, namely T@PCS and AT@PCS, displayed an emergent absorption peak at approximately 1710 cm^−1^, assignable to the characteristic stretching vibration of the ester carbonyl group (C=O) in TA, which constitutes direct spectroscopic evidence of the successful incorporation of TA into the fibrous matrix. The pronounced enhancement of absorption intensity at approximately 1640 cm^−1^ is attributable to the superimposition of vibrational signals arising from the functional groups intrinsic to TA. The attenuation of absorption intensity at approximately 1610 cm^−1^ observed in TA-containing groups reflects the formation of robust intermolecular hydrogen bonds between the polyphenolic hydroxyl groups of TA and the amino groups of QCS, which competitively displaced the pre-existing strong hydrogen-bonding configurations and consequently reorganized the hydrogen-bonding network of the system, corroborating the formation of a dense crosslinked network mediated by TA. The characteristic peak of α-glycosidic linkages at 847 cm^−1^ observed in A@PCS was consistent with that in the reference spectrum of APS, confirming the successful loading of APS into the fibrous matrix. The overall spectral profile of A@PCS bore close resemblance to that of PCS, indicating that APS reorganized the hydrogen bonding network through physical blending in a gentle manner without inducing appreciable alterations in the chemical structure of the composite. The spectral features of AT@PCS represent a superposition of the aforementioned component-specific signals, collectively validating the synergistic construction of shell-layer TA crosslinking modification and core-layer APS loading. Transmission electron microscopy (TEM) imaging of individual AT@PCS fibers revealed a well-defined interface between the core and shell layers with clearly distinguishable electron density contrast, providing direct morphological confirmation of the structural integrity of the core-shell architecture (Fig. S1C). The fluorescence images in Fig. S1D showed red fluorescence in the core layer and green fluorescence in the shell layer, further supporting the successful fabrication of the core-shell structure by coaxial electrospinning.

The force-displacement responses varied among the different nanofibrous membranes (Fig. 1D). A@PCS exhibited the steepest initial increase in force and the highest peak force, followed by abrupt fracture at a relatively low displacement, indicating high resistance to deformation but limited deformability. The TA-crosslinked T@PCS and AT@PCS membranes displayed nonlinear increases in force with increasing displacement. Among the crosslinked membranes, AT@PCS exhibited a higher peak force and greater displacement at break than T@PCS. Following localized water wetting, both membranes remained mechanically testable, while AT@PCS retained a higher peak force and greater displacement at break than T@PCS (Fig. S1E). These results indicate that AT@PCS provides a more favorable balance between load-bearing capacity, deformability, and mechanical integrity under localized wetted conditions. Release was evaluated at pH 5.4 and pH 7.4, representing acidic and near-physiological conditions, respectively, to reflect the dynamic pH of the diabetic wound microenvironment (Fig. 1E). A@PCS and AT@PCS achieved APS loadings of 16.48 ± 0.41% and 7.43 ± 0.20%, with encapsulation efficiencies of 90.6 ± 2.3% and 88.2 ± 2.4%, respectively (Table S1). The TA-crosslinked shell layer not only conferred mechanical toughness upon the fibrous membrane but also exerted a pronounced regulatory effect on the release behavior of core-layer APS. In the shell-free A@PCS, APS underwent a marked burst release, with approximately 76% released at the initial time point, whereas the TA shell in AT@PCS suppressed this burst and enabled a continuous, gradual release over a 144 h period. Notably, AT@PCS displayed a clearly pH-dependent profile, releasing more rapidly and to a greater cumulative extent at pH 7.4 than at pH 5.4, consistent with the partial dissociation of the TA-mediated hydrogen-bonded network as its phenolic hydroxyls become increasingly ionized near neutrality. In contrast, A@PCS exhibited nearly superimposable profiles at the two pH values, indicating that the pH-dependent release behavior originates from the TA shell rather than the polysaccharide matrix. To further analyze the release mechanism, the AT@PCS release data were fitted to the Korsmeyer–Peppas model. The fitting results indicated that APS release was associated with a diffusion-dominated sustained release process, supporting the role of the TA-crosslinked shell in restraining APS outward diffusion from the core layer (Fig. S2). This sustained delivery profile helps maintain the bioactive availability of APS within the dressing application interval and provides a release basis for the subsequent immunomodulatory and tissue-regeneration processes.

To elucidate the influence of TA incorporation on the surface physicochemical properties and interfacial behavior of polysaccharide nanofiber membranes, water contact angle (WCA) measurements revealed a progressive and sequential increase in WCA values from 59.2 ± 6.2° (PCS) to 93.9 ± 4.6° (AT@PCS), indicating a transition from a hydrophilic to a moderately hydrophobic surface (Fig. 1F and G). This transition is intimately associated with the steric shielding imposed by the TA crosslinked network on hydrophilic functional groups of the polysaccharide chains. Furthermore, the characteristic pale-yellow coloration of the TA-containing groups originates from the intrinsic polyphenolic chromophores of TA, visually indicating its incorporation and imparting a close chromatic resemblance to native skin tone, while its abundant phenolic hydroxyl groups provide potential radical-scavenging sites. Dynamic adhesion testing on finger joints demonstrated that AT@PCS maintained stable attachment without delamination even upon finger flexion to 90°, reflecting its capacity to conform to the complex anatomical geometry of dynamic wound sites (Fig. 1H, Fig. S1F). In the moisture retention assay, glycerin (Gl) exhibited the highest moisture retention (11.73 ± 0.28%), whereas AT@PCS showed the highest value among the nanofibrous membranes (7.16 ± 0.63%), comparable to T@PCS (6.83 ± 0.82%); this water-retaining capacity may be associated with hydrophilic groups and hydrogen-bond networks within the membranes (Fig. S3A). In a separate PBS absorption assay, T@PCS and AT@PCS absorbed 458.33 ± 12.66% and 451.00 ± 14.00% PBS after 1 h, respectively, confirming the liquid-absorption capacity of both membranes, whereas PCS and A@PCS were not evaluated because of their disintegration in PBS (Fig. S3B). Notably, moderate surface hydrophobicity did not preclude bulk fluid uptake, as the latter was also governed by the hydrophilic internal components and the porous fibrous architecture. At the functional level, the core-shell architecture integrated complementary functions, with the TA-crosslinked shell contributing to interfacial stability and the APS-loaded core enabling sustained delivery, consistent with the skin-inspired defense-regeneration design. Collectively, AT@PCS combined surface wetting resistance, conformability, moisture retention, and substantial PBS absorption, which may facilitate fluid management while retaining moisture at the wound interface and thereby support moist wound management (Fig. 1I).

### 3.2 Antibacterial efficacy and inhibition of biofilm formation by AT@PCS nanofibers

**Fig. 2:**
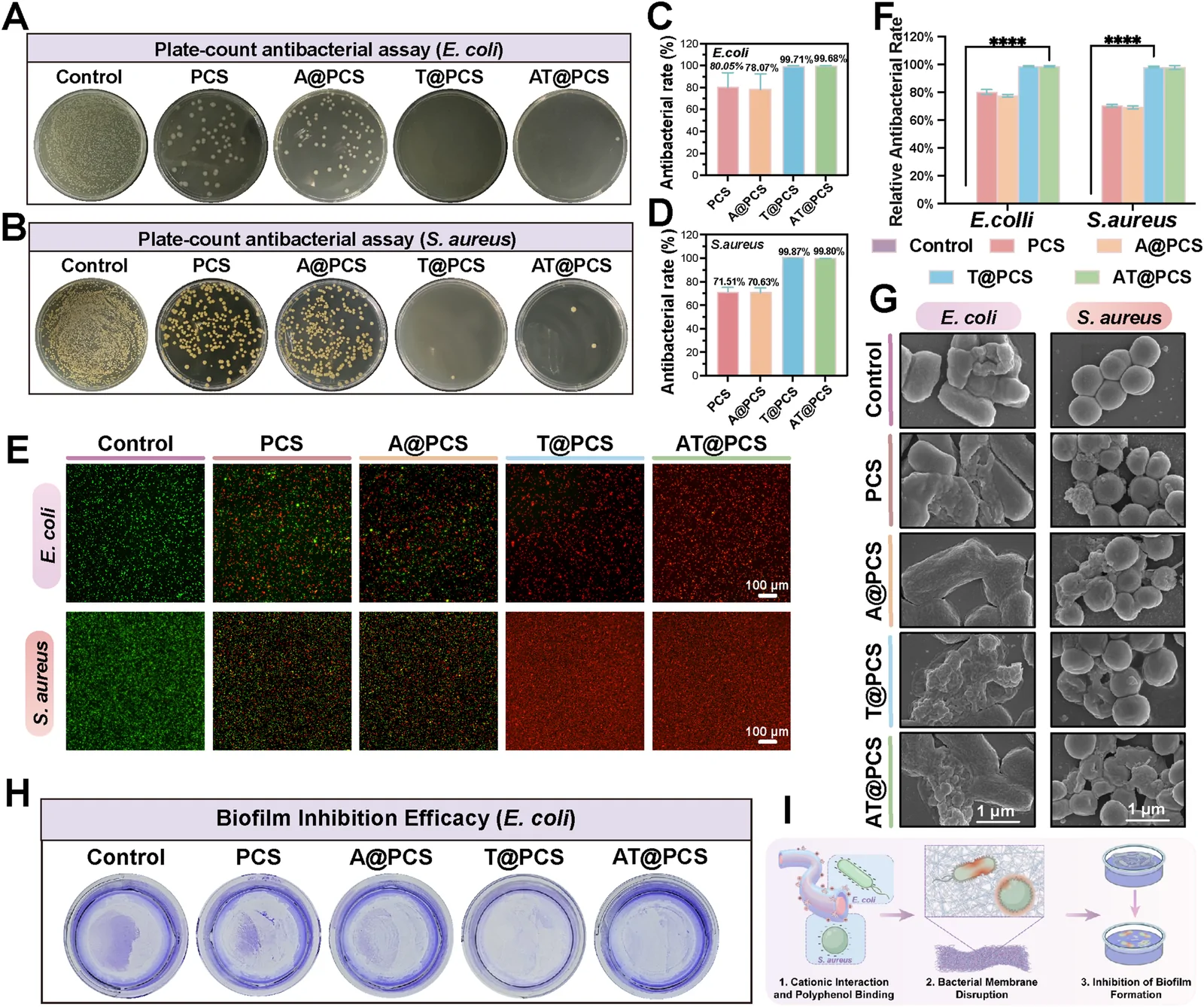
**Antibacterial activity and inhibition of biofilm formation by core-shell polysaccharide nanofiber dressings. (A, B)** Representative bacterial colony images showing the antibacterial activity of PCS, A@PCS, T@PCS, and AT@PCS against *E. coli* and *S. aureus*. **(C, D)** Quantitative analysis of antibacterial rates for PCS, A@PCS, T@PCS, and AT@PCS against *E. coli* and *S. aureus*, respectively. **(E)** Fluorescence microscopy images of bacterial viability assessed by live/dead staining (green: viable bacteria; red: nonviable bacteria; scale bar = 100 μm). **(F)** Relative antibacterial rate determined by live/dead bacterial staining. **(G)** SEM images of *E. coli* and *S. aureus* following the indicated treatments (scale bar = 1 μm). **(H)** Inhibitory effects of various samples on *E. coli* biofilm formation. **(I)** Schematic illustration of the antibacterial process of AT@PCS. (For interpretation of the references to color in this figure legend, the reader is referred to the Web version of this article.)

Persistent infection in diabetic wounds hinders healing; thus, the antibacterial efficacy of the dressings was evaluated. *E. coli* and *S. aureus* were selected as representative Gram-negative and Gram-positive bacterial strains, respectively, to evaluate the *in vitro* antibacterial performance of the dressings across all experimental groups. Representative agar plate images revealed comparable antibacterial efficacy between the PCS and A@PCS groups (Fig. 2A and B), a finding further corroborated by quantitative analysis, which demonstrated that the antibacterial rates of the PCS group against *E. coli* and *S. aureus* were 80.05 ± 13.49% and 71.51 ± 3.67%, respectively, values closely approximating those of the A@PCS group (78.07 ± 14.46% and 70.63 ± 4.21%, respectively) (Fig. 2C and D). These findings collectively indicate that the incorporation of APS did not meaningfully compromise the intrinsic antibacterial performance of the nanofiber system. The incorporation of TA substantially enhanced antibacterial efficacy, with T@PCS and AT@PCS achieving inhibition rates exceeding 99.6% against both bacterial strains. The absence of statistically significant differences between the two groups, coupled with their consistently superior broad-spectrum antibacterial performance, collectively confirms that shell-layer TA constitutes a major component contributing to the observed antibacterial activity. Consistent with this, the free fraction of shell-layer TA was released and reached a plateau within the first few hours (Fig. S5B). Because TA exerts contact-based interfacial antibacterial activity that does not depend on diffusion-limited accumulation, the antibacterial function of the shell is established rapidly during the early phase of application, in line with the front-loaded defensive role assigned to the shell in the temporally coordinated design.

Live/dead staining further confirmed the differences in antibacterial activity among groups. In the control group, both bacterial strains exhibited predominantly green fluorescence, indicative of viable cells. In the PCS group, a discernible attenuation of the green fluorescent signal was observed, suggesting that the cationic properties of QCS conferred a baseline antibacterial effect. Following treatment with AT@PCS, the green fluorescence signal was almost entirely eliminated, with red fluorescence from nonviable bacteria becoming predominant (Fig. 2E and F). The ratio of live to dead bacteria was significantly reduced in the AT@PCS group relative to the control group for both *E. coli* and *S. aureus* (both *p* < 0.001). Because propidium iodide penetrates bacteria only upon loss of membrane integrity, the increased red fluorescence indicated membrane disruption following AT@PCS treatment. This mechanistic interpretation is further corroborated by the membrane structural disruption observed in SEM imaging. SEM examination of bacterial morphology following treatment with AT@PCS membrane discs (Fig. 2G) revealed that *E. coli* and *S. aureus* in the control group maintained intact and fully turgid cellular morphologies. In contrast, *E. coli* cells exhibited pronounced shrinkage and leakage of intracellular contents, while *S. aureus* demonstrated marked cell wall disruption accompanied by surface roughening, collectively reflecting structural deterioration of bacterial cells. The antibacterial effect likely involved the combined action of QCS and TA. The permanent positive charges of QCS may facilitate electrostatic interactions with negatively charged bacterial surfaces, thereby disturbing bacterial membrane integrity [35,36]. TA, as a polyphenolic molecule, may further contribute to antibacterial activity through interactions with bacterial surface proteins and extracellular matrix components [37].

In diabetic infected wounds, bacteria are capable of secreting extracellular polymeric substances (EPS) to initiate biofilm formation at the earliest stages of colonization, whereby pathogens become encapsulated within the EPS matrix and consequently develop pronounced tolerance to antimicrobial agents, rendering diabetic infected wounds refractory to conventional therapeutic intervention. Crystal violet staining revealed biofilm biomass at the bottom of the culture wells in both the control and PCS groups for both *E. coli* and *S. aureus*. In contrast, biofilm biomass was markedly diminished in the T@PCS and AT@PCS treatment groups, with the AT@PCS group exhibiting virtually no biofilm formation (Fig. 2H, Fig. S4). These observations indicate that the combined antibacterial activity arising from the shell-layer TA and QCS not only targets planktonic bacteria but also inhibits bacterial biofilm formation. These results are schematically summarized in Fig. 2I. Together, they suggest that the TA/QCS-modified shell effectively reduces the early bacterial burden, thereby establishing a favorable foundation upon which core-layer APS can mediate subsequent immunomodulation and tissue regeneration.

### 3.3 Biocompatibility and regulation of cellular behaviors *in vitro*

**Fig. 3:**
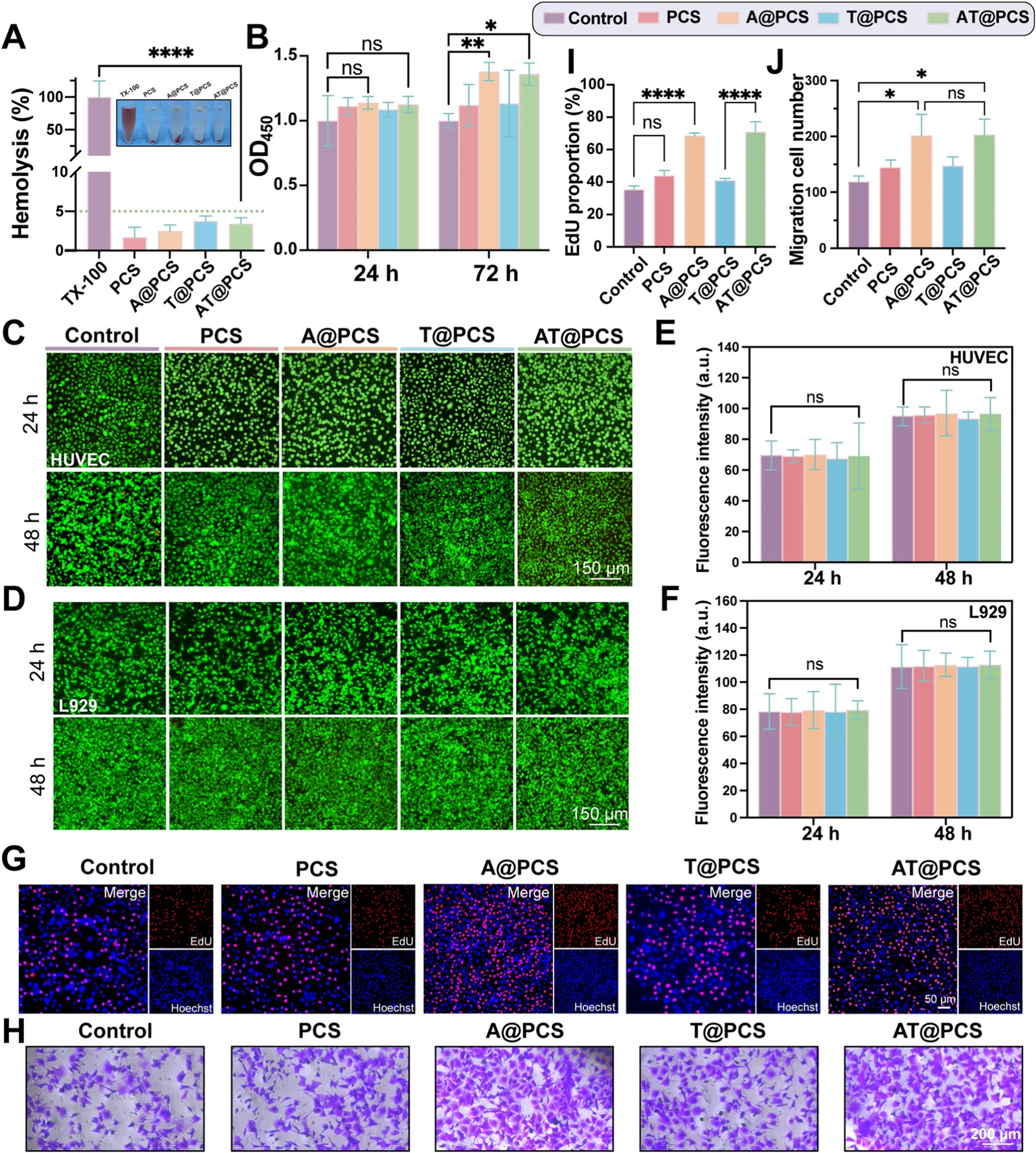
***In vitro*****biocompatibility and cellular behavior modulation of core-shell polysaccharide nanofiber dressings. (A)** Representative photographs of blood samples following co-incubation with distinct nanofiber dressings and corresponding quantitative analysis of hemolytic rates, with Triton X-100 serving as the positive control. **(B)** CCK-8 cell viability analysis of L929 cells following treatment with extracts for 24 and 72 h. **(C, D)** Live/dead fluorescence staining images of HUVECs and L929 cells at 24 and 48 h (green, live cells; red, dead cells; scale bar = 150 μm). **(E, F)** Corresponding quantitative analysis of live/dead cell fluorescence intensities. **(G)** Representative fluorescence images of EdU proliferation staining in L929 cells (scale bar = 50 μm). **(H)** Representative images of the Transwell migration assay in L929 cells (scale bar = 200 μm). **(I)** Quantitative analysis of the proportion of EdU-positive L929 cells. **(J)** Quantitative analysis of the number of migrated L929 cells. (For interpretation of the references to color in this figure legend, the reader is referred to the Web version of this article.)

*In vitro* biosafety was evaluated through hemolysis, CCK-8, and live/dead staining assays. Among these, the hemolysis assay revealed that the hemolytic rates of all dressings ranged from 1.71 ± 1.27% to 3.76 ± 0.65%, remaining substantially below the internationally recognized safety threshold of 5%, thereby confirming the favorable hemocompatibility of all tested dressings (Fig. 3A). Using L929 cells as a model system, cytotoxicity was further evaluated using the CCK-8 assay. At 24 h, cell viability across all experimental groups was comparable to that of the control group, with no statistically significant differences observed (*p* > 0.05). At 72 h, A@PCS and AT@PCS significantly promoted L929 cell proliferation (*p* < 0.01, *p* < 0.05) compared with the control (Fig. 3B). Live/dead staining confirmed no discernible cytotoxicity in any group at 24 and 48 h (Fig. 3C–F).

The proliferation and directional migration of fibroblasts are key cellular processes underlying granulation tissue formation and re-epithelialization. Accordingly, the present study employed EdU proliferation assays and Transwell migration assays to evaluate the effects of dressing extracts derived from each experimental group on the functional properties of L929 cells. As illustrated in Fig. 3G, the A@PCS and AT@PCS groups exhibited markedly stronger EdU-positive fluorescence signals compared with the PCS and T@PCS groups. Quantitative analysis revealed that the proportions of EdU-positive cells in these two groups were 68.55 ± 1.76% and 70.93 ± 6.30%, respectively, both of which significantly exceeded the 35.39 ± 2.13% observed in the control group (*p* < 0.001). These results suggest that APS loading was associated with increased S-phase entry. In contrast, the PCS and T@PCS groups showed no statistically significant difference in EdU-positive cell proportions relative to the control (*p* > 0.05), suggesting that the QCS and TA components themselves exerted no discernible influence on cellular proliferative activity (Fig. 3I). These observations were corroborated by the Transwell migration assay (Fig. 3H). The number of migrated cells in the A@PCS and AT@PCS groups was significantly greater than that in the control group (*p* < 0.05), whereas no statistically significant difference was detected between the PCS or T@PCS groups and the control group (*p* > 0.05). Collectively, these findings indicated that extracts from APS-containing membranes contributed to enhanced fibroblast migration (Fig. 3J). Overall, AT@PCS demonstrated favorable biocompatibility and significantly enhanced fibroblast proliferation and migration *in vitro*.

### 3.4 Hierarchical antioxidant defense and mitochondrial functional protection

Diabetic wounds are characterized by excessive ROS accumulation [1]. Excessive ROS directly attack cellular membrane phospholipids, initiating lipid peroxidation chain reactions, and diffuse into mitochondria, further exacerbating electron leakage and superoxide radical generation, ultimately perpetuating a self-reinforcing vicious cycle of “ROS-induced mitochondrial damage and subsequent ROS regeneration” [38]. This dual oxidative damage, occurring simultaneously at extracellular and intracellular levels, means that antioxidant strategies acting only at the cell surface are insufficient to interrupt the ensuing cascade of cellular injury. Accordingly, based on the functional compartmentalization of AT@PCS, we proposed an integrated therapeutic strategy targeting extracellular oxidative stress, intracellular antioxidant defense, and mitochondrial protection. This section further investigates the radical- and ROS-scavenging activity of shell-layer TA and the association of sustained core-layer APS release with enhanced Nrf2/HO-1-associated antioxidant defense and improved mitochondrial bioenergetic function.

**Fig. 4:**
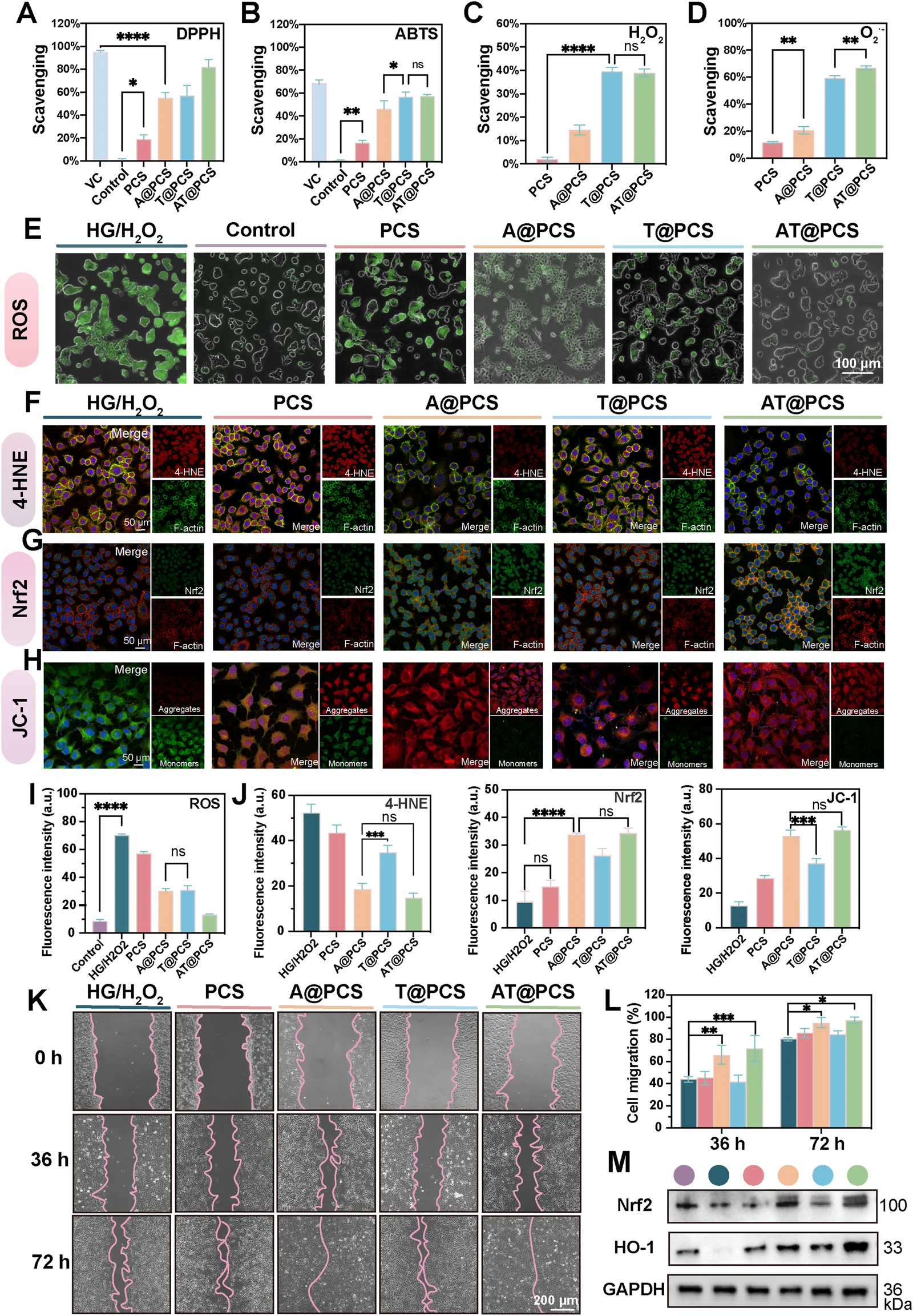
**Core-shell polysaccharide nanofibrous dressings alleviate oxidative stress and preserve mitochondrial function through ROS scavenging. (A-D)** Scavenging efficiencies of the nanofibrous membranes against DPPH radicals, ABTS radical cations, H_2_O_2_, and O_2_^·-^, respectively. **(E)** Fluorescence images of intracellular ROS in HUVECs under HG/H_2_O_2_-induced oxidative stress conditions (scale bar = 100 μm). **(F-G)** Fluorescence staining images of 4-HNE oxidative damage and Nrf2 expression in RAW 264.7 cells under HG/H_2_O_2_-induced oxidative stress conditions (scale bar = 50 μm). **(H)** JC-1 fluorescence staining images of MMP in HUVECs under HG/H_2_O_2_-induced oxidative stress conditions (scale bar = 50 μm). **(I, J)** Quantitative fluorescence intensity analysis of intracellular ROS, 4-HNE, Nrf2, and JC-1 aggregate (red). **(K)** Representative images of the scratch migration assay in L929 cells under oxidative stress conditions (scale bar = 200 μm). **(L)** Quantitative analysis of cell migration rates across all experimental groups. **(M)** Nrf2 and HO-1 protein expression. (For interpretation of the references to color in this figure legend, the reader is referred to the Web version of this article.)

Antioxidant activity was evaluated using DPPH and ABTS radical-scavenging assays together with H_2_O_2_ and O_2_^·-^ scavenging assays. The DPPH assay primarily reflects hydrogen- or electron-donating capacity in an organic medium, whereas the ABTS assay is applicable in both aqueous and organic media, thereby providing a complementary assessment of antioxidant activity. PCS exhibited the lowest DPPH- and ABTS-scavenging activities (18.89 ± 3.89% and 16.58 ± 2.11%, respectively), indicating that the fibrous scaffold itself contributed limited antioxidant activity (Fig. 4A and B and Fig. S6A). The TA-containing T@PCS and AT@PCS membranes exhibited markedly higher H_2_O_2_ scavenging efficiencies (39.19 ± 2.08% and 38.96 ± 1.61%, respectively) than PCS (1.90 ± 0.86%) and A@PCS (14.54 ± 2.14%), with no significant difference between T@PCS and AT@PCS (Fig. 4C). For O_2_^·-^ scavenging, the efficiencies increased from 11.60 ± 0.79% for PCS and 20.60 ± 2.81% for A@PCS to 59.28 ± 1.83% for T@PCS and 66.96 ± 1.50% for AT@PCS, with AT@PCS significantly outperforming T@PCS (Fig. 4D). Collectively, these results identify TA as the predominant antioxidant component, while APS was associated with additional scavenging activity, particularly against O_2_^·-^.

To characterize the temporal antioxidant behavior of the shell, TA release and DPPH-scavenging activity were monitored over time (Fig. S5A and B). TA release increased markedly within 60 min and reached a plateau after 240 min, with only a small fraction of the initially loaded TA being released, suggesting that most TA remained associated with the QCS shell network. Correspondingly, the DPPH-scavenging activities of T@PCS and AT@PCS increased to approximately 88% at 180 min and 98% at 240 min before reaching a plateau after 300 min. This temporal association indicates that antioxidant activity was rapidly established during the early stage of application. In contrast, APS release from the core persisted for up to 120 h (Fig. 1E). Although the TA- and APS-release phases may partially overlap, they establish a functionally staged profile in which early TA-mediated ROS scavenging is complemented by prolonged APS delivery. In practical applications, the core-shell architecture of AT@PCS may therefore provide more durable antioxidant and immunomodulatory support within the wound microenvironment than strategies relying solely on the immediate scavenging activity of TA.

To investigate the capacity of each material group to alleviate oxidative stress and confer mitochondrial protection, a high-glucose/H_2_O_2_ induced oxidative stress model was established by culturing cells in medium containing 25 mM D-glucose and 100 μM H_2_O_2_. Following a 24-h treatment with extracts derived from each experimental group, intracellular ROS levels were measured. The intracellular ROS detection results revealed that the green fluorescence intensity in the HG/H_2_O_2_ group was markedly elevated relative to the unstimulated control group, whereas the AT@PCS group exhibited the weakest green fluorescence signal among the material-treated groups (Fig. 4E). Quantitative analysis revealed that both the A@PCS group (30.41 ± 1.57 AU) and the T@PCS group (31.01 ± 2.97 AU) reduced intracellular ROS levels relative to those of the HG/H_2_O_2_ group, while the AT@PCS group showed a further reduction to 13.09 ± 0.46 AU, with a significantly lower value than those of the other material-treatment groups (*p* < 0.001), demonstrating the most pronounced intracellular ROS scavenging capacity among all groups examined (Fig. 4I).

The level of 4-HNE reflects the extent of lipid peroxidation damage under HG/H_2_O_2_-induced oxidative stress, while Nrf2 expression is associated with the cellular antioxidant response regulated through the Keap1/Nrf2/ARE pathway. As depicted in Fig. 4F, the HG/H_2_O_2_ group exhibited the highest red fluorescence signal for 4-HNE at approximately 52.17 ± 3.88 AU, whereas the T@PCS group demonstrated a reduction to 34.38 ± 3.62 AU. More pronounced attenuation was observed in the A@PCS and AT@PCS groups, which recorded values of 18.56 ± 2.63 AU and 14.72 ± 2.08 AU, respectively, both of which were significantly lower than that of the T@PCS group (*p* < 0.001). Notably, no statistically significant difference was detected between the two APS-loaded groups (*p* > 0.05; Fig. 4J). Although T@PCS demonstrated greater activity in extracellular free radical scavenging relative to PCS and A@PCS, its protective efficacy against cellular membrane lipid peroxidation was markedly inferior to that of A@PCS. Fluorescence staining of Nrf2 (Fig. 4G) revealed that Nrf2 intensity in RAW 264.7 cells was significantly higher in the A@PCS, T@PCS, and AT@PCS groups than in the HG/H_2_O_2_ stimulated control group (*p* < 0.001). The A@PCS (33.67 ± 0.64 AU) and AT@PCS (34.36 ± 1.85 AU) groups reached comparable levels, both exceeding T@PCS (26.22 ± 2.57 AU), and all markedly above the control (9.54 ± 3.76 AU). The absence of a significant difference between the A@PCS and AT@PCS groups (*p* > 0.05) suggests that APS loading was a major contributor to Nrf2 upregulation (Fig. 4J). The elevated Nrf2 expression paralleled the reduced intracellular ROS and 4-HNE levels in these groups, supporting an association between enhanced endogenous antioxidant defense and reduced lipid peroxidation. To further characterize the Nrf2-associated antioxidant response and assess its downstream effector, the protein levels of Nrf2 and HO-1, a canonical Nrf2-regulated antioxidant enzyme, were examined by Western blotting. Consistent with the Nrf2 immunofluorescence results, A@PCS and AT@PCS significantly increased Nrf2 protein expression and concomitantly elevated HO-1 levels compared with the HG/H_2_O_2_ group (Fig. 4M and Fig. S7). These findings support the coordinated upregulation of the Nrf2/HO-1 antioxidant axis.

Mitochondria play a central role in energy metabolism and redox homeostasis, and maintenance of the MMP is critical for mitochondrial function and cell survival. Oxidative stress can induce MMP depolarization and mitochondrial damage, potentially activating mitochondria-mediated apoptotic signaling pathways [39]. JC-1 staining (Fig. 4H) revealed robust red fluorescence in cells treated with the fibrous membrane extracts, indicating a high degree of MMP integrity and functional preservation. In contrast, the HG/H_2_O_2_-treated control group exhibited a marked reduction in red fluorescence accompanied by a corresponding increase in green fluorescence, reflecting oxidative stress-induced dissipation of membrane potential and consequent impairment of mitochondrial function. Moreover, the degree of recovery observed in the T@PCS group was significantly lower than that of both the A@PCS and AT@PCS groups (*p* < 0.001). Quantitative analysis of the JC-1 fluorescence intensity (Fig. 4J) provided further corroboration of these findings. Consistent results were obtained in RAW 264.7 cells (Fig. S6B and C), collectively demonstrating that AT@PCS effectively preserves MMP stability and attenuates oxidative stress-induced mitochondrial damage. Consistent with the changes in MMP reflected by JC-1 staining, HG/H_2_O_2_-induced oxidative stress markedly impaired cellular ATP production, indicating disrupted mitochondrial energy metabolism. After treatment with material extracts, both the A@PCS and AT@PCS groups effectively restored intracellular ATP levels, whereas T@PCS produced only a limited improvement (Fig. S6D). In contrast, APS-containing groups exhibited more pronounced recovery of ATP production, further indicating that core-layer APS plays a key role in maintaining mitochondrial function.

To further determine whether mitochondrial function was restored at the level of respiratory metabolism, Seahorse cellular energy metabolism analysis was performed to measure oxygen consumption rate (OCR) and extracellular acidification rate (ECAR). The OCR profiles and corresponding quantitative results showed that HG/H_2_O_2_-induced oxidative stress markedly impaired mitochondrial respiratory function (Fig. S6E), as reflected by pronounced decreases in both basal respiration (Fig. S6F) and maximal respiration (Fig. S6G). PCS treatment produced only limited improvement, whereas the APS-containing A@PCS and AT@PCS groups significantly improved both basal and maximal respiratory capacities. Among these groups, AT@PCS exhibited the most prominent recovery effect and outperformed T@PCS. ECAR analysis (Fig. S6H) further showed that HG/H_2_O_2_ stimulation markedly increased glycolysis (Fig. S6I) and glycolytic capacity (Fig. S6J), indicating a compensatory metabolic shift toward glycolysis following oxidative stress-induced impairment of mitochondrial respiration. After treatment with material extracts, this enhanced glycolytic tendency was attenuated to varying degrees. PCS exerted only a limited effect, whereas A@PCS, T@PCS, and AT@PCS reduced both glycolysis and glycolytic capacity. Notably, AT@PCS produced the most pronounced reduction in glycolytic capacity, suggesting that it improves mitochondrial respiratory capacity and alleviates oxidative stress-induced compensatory glycolytic shift. Together with the JC-1, ATP, and Seahorse results, these findings indicate that AT@PCS helps maintain MMP, improve ATP production, and support mitochondrial bioenergetic function while attenuating the compensatory glycolytic shift induced by oxidative stress.

Oxidative stress has been demonstrated to impair cellular migratory capacity through the disruption of mitochondrial function and the perturbation of actin cytoskeletal dynamic remodeling [40]. The migratory capacity of L929 cells under HG/H_2_O_2_-induced oxidative stress was assessed using a scratch assay. As illustrated in Fig. 4K, the migration rates of the PCS and T@PCS groups at 72 h were not significantly different from that of the HG/H_2_O_2_ group (*p* > 0.05), whereas the A@PCS and AT@PCS groups demonstrated markedly elevated migration rates in comparison to the control (*p* < 0.05, Fig. 4L). In contrast, the restoration of migratory function closely paralleled the APS-associated improvement in mitochondrial function, suggesting that mitochondrial bioenergetic recovery may contribute to the reconstitution of L929 cell migratory capacity under oxidative stress. Together, these findings show that AT@PCS integrates rapid TA-associated ROS scavenging with prolonged APS delivery. This coordinated antioxidant strategy reduced cellular oxidative damage, preserved mitochondrial function, and supported cell migration under oxidative stress.

### 3.5 AT@PCS regulates macrophage polarization and promotes angiogenic activity

**Fig. 5:**
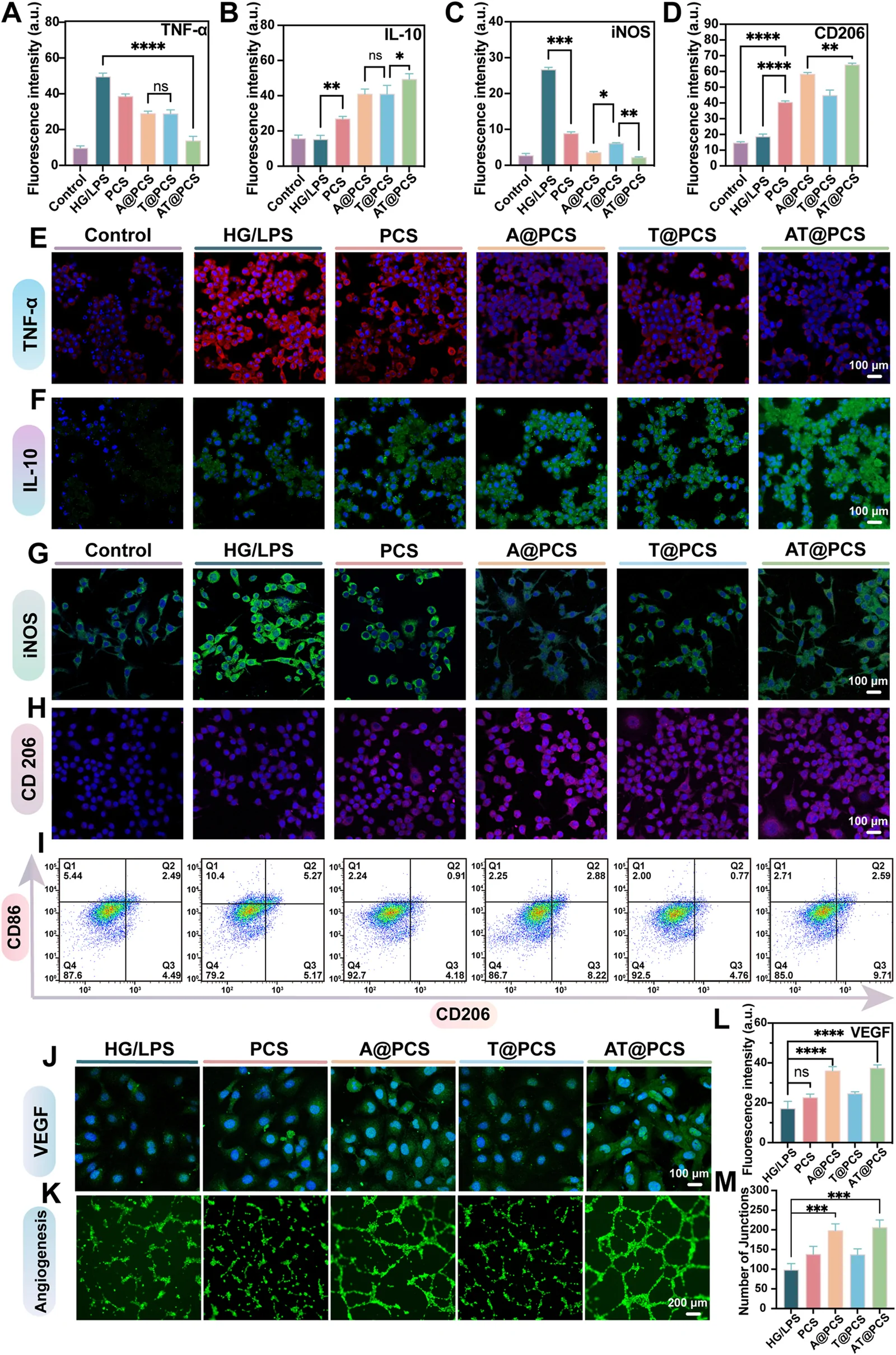
**Core-shell polysaccharide nanofiber dressings promote macrophage M2 polarization and enhance angiogenic capacity. (A-D)** Quantitative analysis of TNF-α, IL-10, iNOS, and CD206 fluorescence intensity in RAW 264.7 macrophages under HG/LPS-induced conditions. **(E, F)** Immunofluorescence staining images of TNF-α and IL-10 (scale bar = 100 μm). **(G, H)** Immunofluorescence staining images of iNOS and CD206 (scale bar = 100 μm). **(I)** Flow cytometric analysis of CD86^+^CD206^-^ and CD86^−^CD206^+^ macrophage populations. **(J, L)** Immunofluorescence staining images and quantitative analysis of VEGF fluorescence intensity in HUVECs (scale bar = 100 μm). **(K, M)***In vitro* tube formation assay images and quantitative analysis of tubular network junction counts in HUVECs (scale bar = 200 μm).

Because oxidative stress is closely associated with macrophage polarization, the effects on macrophage phenotype were further evaluated [41]. Furthermore, M2 macrophages can activate VEGF-associated signaling pathways through paracrine mechanisms, thereby promoting endothelial cell proliferation and angiogenesis and facilitating the transition of diabetic wounds from a state of chronic inflammation toward tissue regeneration [42]. Within an HG/LPS-induced RAW 264.7 macrophage model, the regulatory capacity of leachates derived from each dressing group to modulate macrophage immunophenotypic remodeling was systematically evaluated through the detection of both pro-inflammatory and anti-inflammatory cytokines alongside phenotypic markers (Fig. 5A–D). TNF-α and IL-6 were used as markers of the pro-inflammatory response. Immunofluorescence quantitative analysis (Fig. 5A and E) revealed that the fluorescence intensity of TNF-α in the HG/LPS group was significantly elevated relative to the unstimulated control group (*p* < 0.001), confirming the successful establishment of a pro-inflammatory microenvironment. Following treatment with each respective group, TNF-α expression in the PCS group remained at a similarly high level, suggesting that the polysaccharide matrix scaffold alone possesses limited anti-inflammatory regulatory capacity. In contrast, TNF-α levels in the A@PCS and T@PCS groups were significantly downregulated compared to the HG/LPS group (*p* < 0.001), while TNF-α expression in the AT@PCS group was further reduced to levels approximating those of the unstimulated control, indicating that the combined effects of TA and APS more effectively suppress the excessive activation of pro-inflammatory signaling. Consistently, IL-6 expression showed a similar pro-inflammatory trend, with marked upregulation after HG/LPS stimulation and significant attenuation after treatment with A@PCS, T@PCS, and AT@PCS, among which AT@PCS produced the most pronounced inhibitory effect (Fig. S8A and B). Conversely, IL-10 expression was markedly increased in the A@PCS and AT@PCS groups (Fig. 5B and F). Together with the reduced TNF-α and IL-6 levels, this finding indicates a shift from a pro-inflammatory to the reparative, anti-inflammatory microenvironment. To further characterize macrophage phenotypic remodeling, the expression of the M1-associated marker iNOS and the M2-associated markers CD206 and CD163 was examined (Fig. 5C, D, G, H, and Figs. S8A, C). HG/LPS stimulation markedly increased iNOS expression, whereas CD206 and CD163 expression remained relatively low, indicating a shift toward a pro-inflammatory macrophage phenotype. After treatment with material extracts, iNOS expression was downregulated to varying degrees, whereas CD206 and CD163 expression were restored, with the AT@PCS group showing the most pronounced regulatory effect. At the transcriptional level, qPCR analysis showed that HG/LPS stimulation markedly increased the expression of the pro-inflammatory gene IL-1β, while the expression of reparative macrophage-associated genes, including Arg-1, Ym1, and TGF-β, remained at relatively low levels (Fig. S8D–G). Treatment with material extracts shifted this transcriptional profile to varying degrees, as evidenced by reduced IL-1β expression and increased Arg-1, Ym1, and TGF-β expression, with A@PCS and AT@PCS exhibiting stronger effects. Together, the coordinated reduction of pro-inflammatory cytokines and M1-associated markers, along with the upregulation of anti-inflammatory cytokine expression and reparative macrophage-associated markers at both protein and mRNA levels, demonstrates that AT@PCS effectively suppresses HG/LPS-induced pro-inflammatory activation and promotes macrophage remodeling toward a reparative phenotype.

To further validate macrophage phenotypic remodeling at the cell population level, flow cytometry was used to analyze M1-and M2-associated populations (Fig. 5I). The proportion of CD86^−^CD206^+^ cells (Q3) was significantly increased in the AT@PCS group compared with the HG/LPS group (*p* < 0.001), whereas the HG/LPS-induced increase in CD86^+^CD206^-^ cells (Q1) was markedly attenuated after treatment (Fig. S8H and I). These results corroborated the immunofluorescence and qPCR findings, indicating that AT@PCS favored macrophage phenotypic remodeling toward a reparative state. The stronger effects observed in the APS-containing groups suggest that APS loading contributed substantially to this response. Together with extracellular ROS scavenging by shell-layer TA, sustained APS delivery may support mitochondrial bioenergetic recovery, thereby providing a favorable metabolic context for this phenotypic remodeling [43].

Excessive ROS can impair VEGF-mediated signaling and suppress endothelial cell migration and tube formation, thereby compromising angiogenesis in diabetic wounds [44]. Given the antioxidant and immunomodulatory effects described above, AT@PCS may provide a favorable microenvironment for endothelial angiogenesis. To evaluate the pro-angiogenic effects of AT@PCS, the angiogenic capacity of extracts derived from each dressing group was assessed using VEGF immunofluorescence staining and *in vitro* tube formation assays (Fig. 5J–M). As illustrated in Fig. 5J and L, VEGF fluorescence intensity in both the A@PCS and AT@PCS groups was significantly elevated relative to the HG/LPS and PCS groups (*p* < 0.001), whereas no statistically significant difference was observed between the A@PCS and AT@PCS groups (*p* > 0.05), indicating that APS loading was a major contributor to VEGF upregulation in endothelial cells. The *in vitro* tube formation assay provided further functional validation of this effect (Fig. 5K and M). The number of tubular network junctions in both the A@PCS and AT@PCS groups was significantly greater than that in the HG/LPS and T@PCS groups (*p* < 0.001), with no significant difference between the A@PCS and AT@PCS groups, further supporting a major contribution of APS to the pro-angiogenic activity of the material. To further determine whether macrophage regulation by AT@PCS could indirectly support angiogenesis through paracrine signaling, HUVECs were cultured with conditioned media (CM) collected from RAW 264.7 macrophages subjected to different treatments. VEGF immunofluorescence staining showed that CM from HG/LPS-stimulated macrophages reduced VEGF expression in HUVECs, whereas CM from A@PCS- and AT@PCS-treated macrophages significantly restored VEGF fluorescence intensity, with no significant difference between these two groups (Fig. S9A and B). Consistently, tube formation analysis showed that CM from A@PCS- and AT@PCS-treated macrophages markedly enhanced endothelial network formation, as reflected by increased junction and mesh numbers, whereas T@PCS showed a more limited effect (Figs. S9A, C, D). Together, these results indicate that APS-containing dressings promote endothelial angiogenic activity both through direct effects on HUVECs and through macrophage-mediated pro-angiogenic paracrine signaling.

### 3.6 *In vivo* evaluation of diabetic wound healing and tissue regeneration

**Fig. 6:**
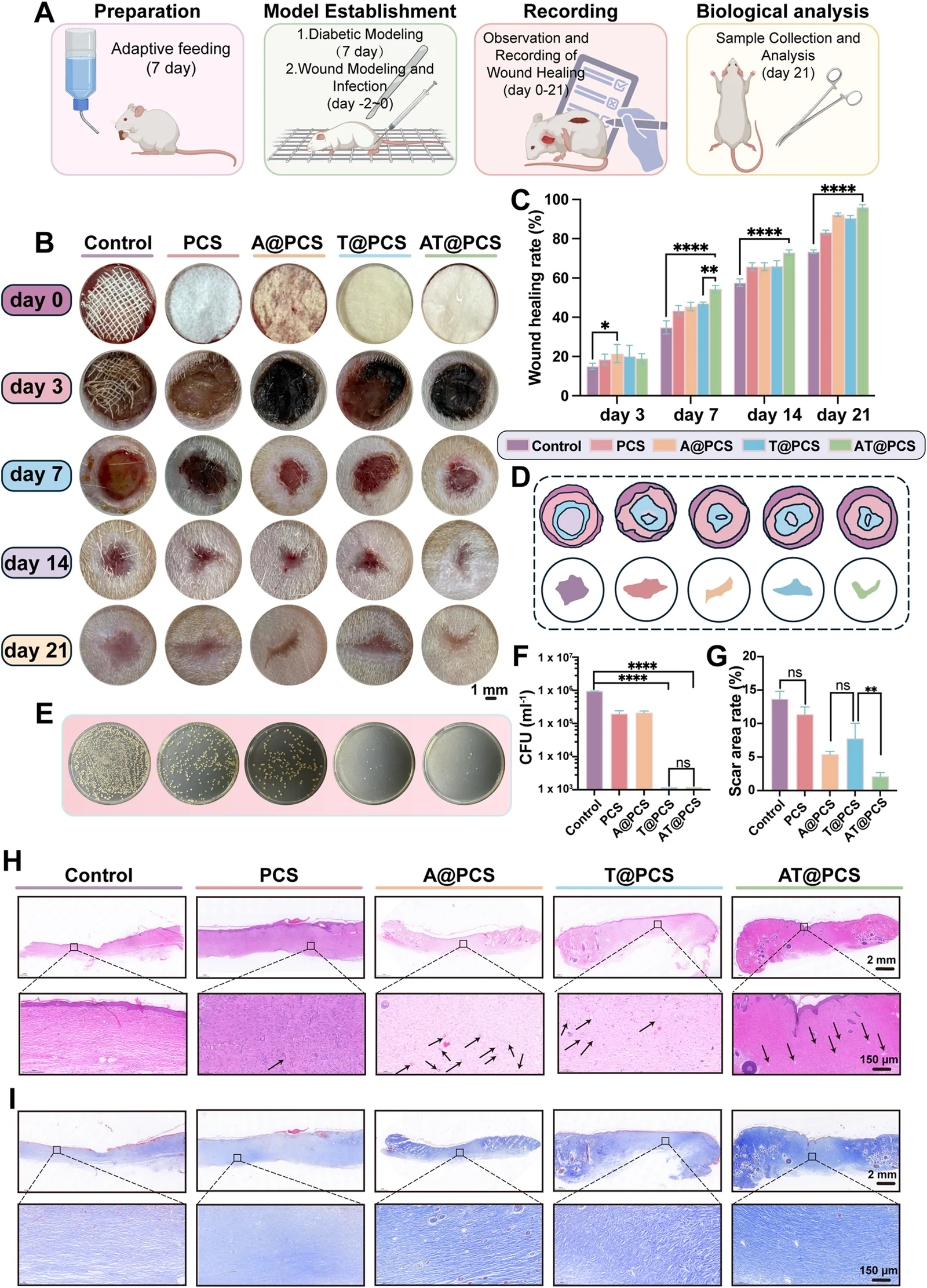
**Evaluation of core-shell polysaccharide nanofiber dressings in promoting wound healing and tissue regeneration in infected diabetic wounds. (A)** Schematic overview of the experimental workflow, encompassing four sequential stages: adaptive housing (7 days), STZ-induced diabetic model establishment (7 days), full-thickness infected wound creation (day −2 to 0), and longitudinal wound observation with tissue analysis at designated time points (days 3, 7, and 21). **(B)** Gross wound images of each group captured on days 0, 3, 7, 14, and 21 (scale bar = 1 mm), and **(C)** corresponding quantitative statistics of wound closure rates. **(D)** Stacked schematic representations of wound area at each time point. **(E)** Agar plate culture images of bacterial colonies recovered from wound exudates on day 3 post-treatment, along with **(F)** the corresponding CFU quantification. **(G)** Quantitative analysis of scar area as a percentage of the original wound area at day 21. **(H)** H&E staining images of wound tissue at day 7 (scale bars = 2 mm for upper panels, 150 μm for lower panels), with black arrows indicating newly formed blood vessels. **(I)** Masson's trichrome staining images at day 7 (scale bars = 2 mm for upper panels, 150 μm for lower panels), with blue regions denoting collagen deposition. (For interpretation of the references to color in this figure legend, the reader is referred to the Web version of this article.)

Based on the complementary shell- and core-mediated effects observed *in vitro*, the therapeutic efficacy of AT@PCS was further evaluated in an STZ-induced diabetic rat model of infected full-thickness wounds (Fig. 6A). Before dressing application, representative photographs obtained 2 days after infection (treatment day 0) documented the baseline wound status in each group (Fig. S10A). Wound images (Fig. 6B) and quantitative analysis of wound closure (Fig. 6C) demonstrated that the AT@PCS group exhibited the greatest reduction in wound area. Wound contour tracing results (Fig. 6D) further illustrated the progression of wound closure, revealing that AT@PCS significantly accelerated wound closure, reaching 71.7 ± 4.4% by day 14 versus 54.8 ± 3.4% in the control group. By day 21, the wound closure rate reached 97.3 ± 1.6% in the AT@PCS group, whereas substantial unclosed areas remained in the control group (*p* < 0.001). Together with the *in vitro* evidence of enhanced cell proliferation and migration, these findings suggest that the combined effects of TA and APS may have contributed to the accelerated wound closure observed with AT@PCS. Bacterial culture results from wound specimens (Fig. 6E and F) revealed that the control and PCS groups harbored substantially elevated bacterial burdens, indicative of limited antimicrobial capacity, whereas colony counts in both the T@PCS and AT@PCS groups fell below the limit of detection, demonstrating markedly superior antibacterial efficacy relative to the PCS and A@PCS groups (*p* < 0.001). This finding is consistent with the antimicrobial contribution of TA in the shell layer, which markedly reduced the wound bacterial burden and thereby created a more favorable environment for subsequent tissue repair.

Building upon these observations, a comparative assessment of scar area across groups at day 21 (Fig. 6G) revealed that the AT@PCS group exhibited the lowest scar-area ratio, which was significantly lower than those of the control, PCS, and T@PCS groups. Together with the *in vitro* findings that APS-containing formulations favored reparative macrophage remodeling, attenuated pro-inflammatory signaling, and increased VEGF expression, the reduced scar-area ratio suggests that the combined effects of TA and APS contributed to reduced scar formation and improved tissue repair. Reparative macrophages may limit fibrosis and support angiogenesis through paracrine mediators [45]. Histological analysis corroborated the superior regenerative outcomes of AT@PCS. H&E staining suggested reduced inflammatory cell infiltration in the AT@PCS group at day 3, with a lower semi-quantitative infiltration score than that in the control group (1.00 ± 1.00 vs. 2.67 ± 0.29; Fig. S10B and C). This early histological trend, together with the antibacterial findings, temporally coincided with the rapid initial release of shell-layer TA and the early antioxidant activity observed *in vitro* (Fig. S5A and B). In a separate subcutaneous implantation assay, AT@PCS underwent rapid and progressive *in vivo* degradation, with cumulative mass loss increasing from 50.62 ± 5.68% on day 1 to 92.77 ± 6.47% by day 4 (Fig. S10D). Histological examination at day 7 revealed that control wounds retained prominent inflammatory features, with limited granulation tissue and few vascular structures. In contrast, AT@PCS-treated wounds exhibited more abundant vascular structures and increased fibroblast-like cellularity, consistent with progression toward the proliferative phase (Fig. 6H). These reparative changes observed on day 7 were consistent with the downstream effects of prolonged APS delivery following the early antibacterial and anti-inflammatory phase. A well-developed neovascular network contributes to improved local oxygen supply and nutrient delivery within the wound bed, thereby providing the requisite microenvironmental support for subsequent collagen remodeling and tissue regeneration. Epidermal thickness analysis further demonstrated that the AT@PCS group attained an epidermal thickness of 79.94 ± 5.75 μm by day 7, significantly exceeding those of all other groups (Fig. S10E). Masson's trichrome staining demonstrated that the AT@PCS group achieved the highest collagen deposition (72.40 ± 2.27%, Fig. S10E), accompanied by more densely and regularly arranged collagen fibers, indicating more advanced extracellular matrix remodeling (Fig. 6I).

### 3.7 *In vivo* effects of inflammation, angiogenesis, and collagen remodeling by AT@PCS

**Fig. 7:**
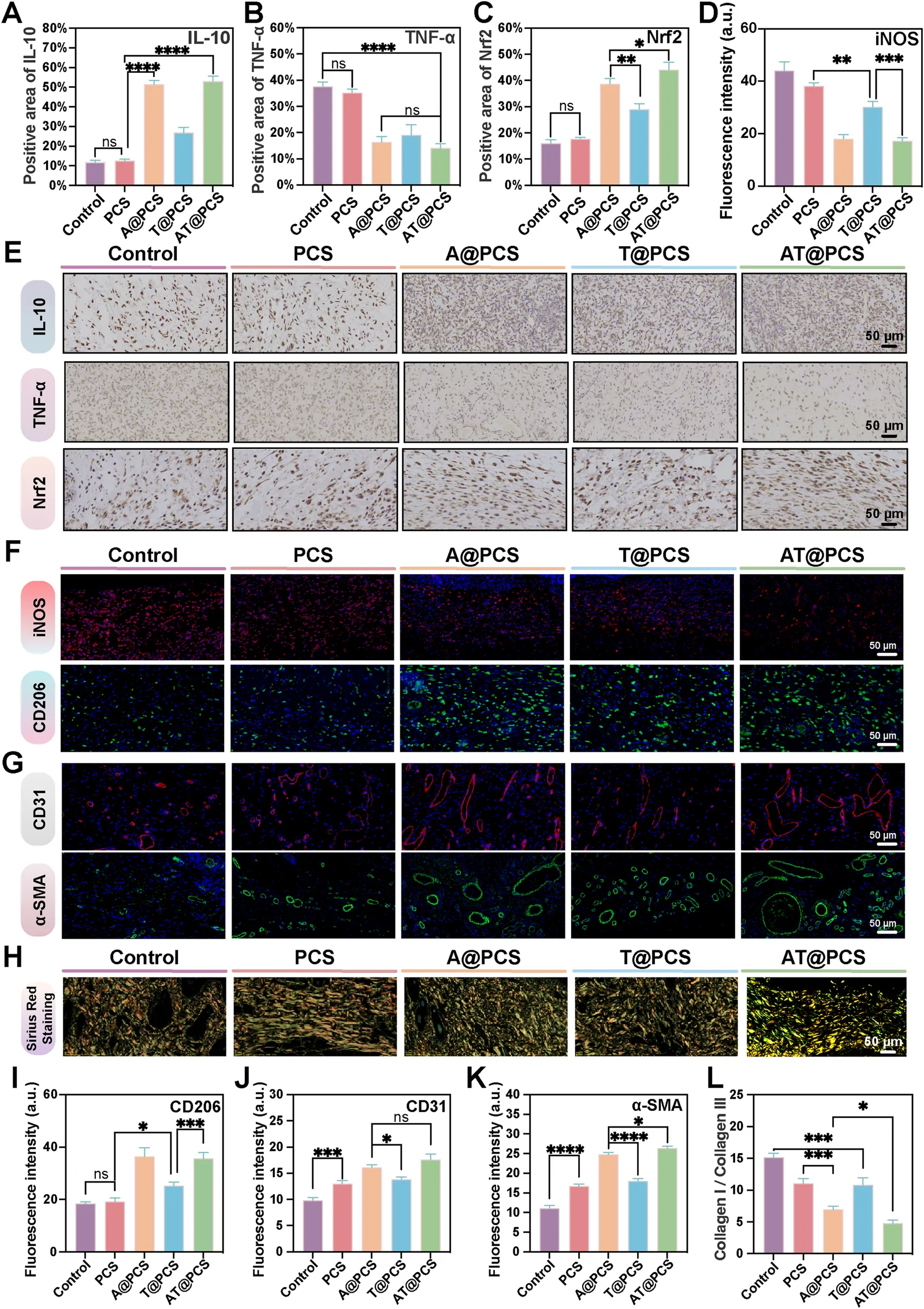
***In vivo*****effects of AT@PCS on inflammation, angiogenesis, and collagen remodeling. (A, B)** Quantitative analysis of the positive staining area for the anti-inflammatory mediator IL-10 and the pro-inflammatory cytokine TNF-α in wound tissues. **(C)** Quantitative analysis of the positive staining area for Nrf2. **(D)** Quantitative analysis of iNOS fluorescence intensity. **(E)** Corresponding immunohistochemical staining images for IL-10, TNF-α, and Nrf2 (scale bar = 50 μm). **(F)** Immunofluorescence staining images of iNOS and CD206 (scale bar = 50 μm). **(G)** Immunofluorescence staining images of CD31 and α-SMA (scale bar = 50 μm). **(H)** Sirius Red polarized light staining images (scale bar = 50 μm). **(I)** Quantitative analysis of CD206 fluorescence intensity. **(J, K)** Quantitative analysis of CD31 and α-SMA fluorescence intensity. **(L)** Quantitative analysis of the collagen I/III ratio derived from Sirius Red staining. (For interpretation of the references to color in this figure legend, the reader is referred to the Web version of this article.)

To further investigate the *in vivo* mechanisms, markers associated with inflammation, oxidative stress regulation, and angiogenesis were evaluated on day 7, whereas collagen remodeling was assessed on day 21. Fig. 7E presents the immunohistochemical staining results for IL-10, TNF-α, and Nrf2. As an anti-inflammatory and tissue repair mediator, elevated IL-10 expression is associated with an anti-inflammatory and reparative wound microenvironment. In contrast, elevated expression of the prototypical pro-inflammatory cytokine TNF-α indicates that the wound remains in a persistent inflammatory state unfavorable for transition toward the proliferative phase. Upon activation, Nrf2 translocates to the nucleus and upregulates antioxidant enzymes such as HO-1, thereby attenuating ROS-mediated cellular damage and suppressing pro-inflammatory signaling. Nrf2 expression thus provides an indicator of the endogenous antioxidant response [46]. Statistical analysis revealed that the AT@PCS group exhibited the highest IL-10 expression (*p* < 0.001, Fig. 7A), the lowest TNF-α expression (Fig. 7B), and likewise the highest Nrf2 expression among all experimental groups (Fig. 7C). Notably, the elevated Nrf2 in the AT@PCS group significantly exceeded that of the A@PCS and T@PCS groups (*p* < 0.05), suggesting that the combined incorporation of TA and APS elicited a stronger tissue-level Nrf2-associated antioxidant response than either component alone. In parallel, *in vitro* Western blotting demonstrated coordinated upregulation of Nrf2 and its downstream antioxidant enzyme HO-1 in the A@PCS and AT@PCS groups (Fig. 4M and Fig. S7A and B), providing complementary molecular evidence for an enhanced Nrf2/HO-1-associated antioxidant response. These findings are consistent with complementary roles for rapid TA-associated ROS scavenging and sustained APS delivery. TA may reduce extracellular oxidative stress, whereas sustained APS delivery may support intracellular antioxidant regulation [47]. The concurrent increase in IL-10 and Nrf2 alongside the decrease in TNF-α indicates attenuation of inflammatory and oxidative stress and progression toward reparative remodeling. Consistent with the *in vitro* macrophage polarization results, immunofluorescence staining of iNOS and CD206 in wound tissues further showed that A@PCS and AT@PCS markedly reduced the expression of the M1-associated marker iNOS (Fig. 7D) while increasing the expression of the M2-associated marker CD206 (Fig. 7F and I). Together, the coordinated changes in inflammatory cytokines, Nrf2-associated antioxidant defense, and macrophage polarization-associated markers support the ability of AT@PCS to alleviate the chronic inflammatory and oxidative stress microenvironment of diabetic wounds.

The attenuation of inflammation may provide a more permissive microenvironment for angiogenesis and subsequent vessel maturation [48]. CD31 was used to identify endothelial microvessels, whereas perivascular α-SMA-positive mural cell coverage was used to assess microvessel maturation. Immunofluorescence staining of wound tissues on day 7 showed more abundant CD31-positive vascular structures and greater perivascular α-SMA coverage in the A@PCS and AT@PCS groups (Fig. 7G, J, K). Vessel-level quantification further showed that the microvessel density (MVD) increased from 15.95 ± 0.54 vessels mm^−2^ in the control group to 25.52 ± 3.11 and 27.38 ± 0.68 vessels mm^−2^ in the A@PCS and AT@PCS groups, respectively, with no significant difference between the two APS-containing groups (Fig. S11A). Similarly, the microvessel pericyte coverage index (MPI) increased from 37.83 ± 1.18% in the control group to 48.54 ± 3.71% and 48.08 ± 4.20% in the A@PCS and AT@PCS groups, respectively, again with no significant difference between A@PCS and AT@PCS (Fig. S11B). These findings indicate that APS-containing dressings increased MVD and perivascular mural cell coverage, providing structural evidence for enhanced angiogenesis and vessel maturation. The comparable MVD and MPI values between A@PCS and AT@PCS suggest that APS loading was the major contributor to these vascular effects, whereas the TA-containing shell may provide complementary antibacterial and antioxidant support within the wound microenvironment.

ECM composition and architecture are important determinants of scar formation. Sirius Red staining under polarized light revealed a more regularly organized collagen architecture in AT@PCS-treated wounds on day 21 (Fig. 7H). Quantitative analysis showed that the AT@PCS group exhibited the lowest collagen I/III ratio (4.77 ± 0.52), which was significantly lower than that of all other groups (Fig. 7L). The lower collagen I/III ratio indicates that type I collagen remained predominant, but its predominance over type III collagen was reduced, reflecting a greater relative proportion of type III collagen. Together with the reduced scar area on day 21, this finding supports an ECM remodeling profile associated with reduced scar formation in AT@PCS-treated wounds.

## 4 Discussion

The core-shell structural design of AT@PCS was validated across the release, antibacterial, antioxidant, and *in vitro* and *in vivo* experiments. The front-loaded release of shell-layer TA and the rapid establishment of antioxidant scavenging within hours, together with the sustained release of core-layer APS over 144 h, defined a temporal sequence that was further reflected *in vivo* as a stage-dependent progression from early antibacterial and anti-inflammatory action toward subsequent immunomodulation and tissue repair. This temporal logic is grounded in a deeper biological rationale, as ROS do not play a uniformly deleterious role in wound healing. Moderate ROS generation during the early phase fulfills indispensable physiological functions in pathogen elimination and immune cell recruitment, whereas excessive and sustained ROS drive chronic inflammation and progressive tissue injury [49]. An optimal intervention should therefore modulate ROS in a temporally coordinated manner rather than eliminate them indiscriminately [50], a goal supported by AT@PCS. The shell components first establish a favorable therapeutic window for infection control, while the core-layer APS subsequently helps attenuate sustained intracellular oxidative stress. This stratified arrangement reflects a skin-inspired functional organization, in which the shell provides early barrier and defense functions, whereas the core supports sustained regulation of cellular bioenergetics and tissue homeostasis.

Core-shell nanofibers reported over the past two years have predominantly employed poly(lactic acid), poly(lactic-co-glycolic acid), or poly(ε-caprolactone) as structural scaffolds [51,52], with antibacterial and pro-reparative agents separately incorporated into the core and shell layers. These systems have demonstrated favorable antibacterial, antioxidant, and tissue-repair effects. In contrast, AT@PCS uses natural polysaccharides as its primary matrix components. Physical crosslinking with TA improves the wet-state stability and conformability of the shell [30,32], while TA/QCS provides early antibacterial and extracellular antioxidant effects [31]. Sustained release of APS from the core is associated with the alleviation of intracellular oxidative stress and immunomodulation [53]. Although the long-term water resistance of this polysaccharide fibrous membrane may be inferior to that of synthetic polymer systems, AT@PCS integrates material performance with biological activity without incorporating antibiotics [52]. Beyond evaluating wound healing and angiogenesis, this study further examined its effects on extracellular and intracellular antioxidant regulation, mitochondrial energy metabolism, and macrophage-endothelial cell interactions.

The antibacterial activity of the TA/QCS-containing shell may be associated with the combined physicochemical contributions of cationic QCS and polyphenolic TA. Existing literature has documented the limited duration of antibacterial efficacy associated with strategies employing QCS or polyphenolic compounds alone [54], whereas AT@PCS integrates both components through co-blending and immobilization within the shell layer, which may allow the antibacterial constituents to retain interfacial activity as the fibrous matrix progressively degrades. This contact-active antibacterial process may be mediated, at least in part, by cationic electrostatic interactions and polyphenol-mediated bacterial surface interactions, thereby potentially enhancing antibacterial efficacy and supporting infection control in diabetic wounds [55]. It is worth noting that strategies relying exclusively on ROS-mediated antibacterial activity, while capable of achieving highly efficient pathogen elimination during the early stage of infection, may paradoxically impede tissue regeneration during the intermediate and late phases owing to the sustained accumulation of ROS [56]. In AT@PCS, the antibacterial activity of TA does not rely on sustained ROS generation, allowing pathogen clearance without introducing additional oxidative burden. By reducing the early bacterial burden, TA may reduce the need for host cells to mount a sustained ROS-mediated response against infection, thereby reducing the infection-associated oxidative load at its source. Building on this, the gradual release of core-layer APS was associated with activation of the Nrf2 pathway and further suppression of residual intracellular oxidative stress. Acting sequentially in time, the two components not only synergistically promote healing but also progressively lower cellular oxidative stress and establish a microenvironment more favorable for the restoration of cellular function [57].

An observation warranting deeper discussion from the *in vitro* experiments is that the TA group, which exhibited predominantly extracellular ROS-scavenging activity, demonstrated markedly less improvement in 4-HNE accumulation and MMP relative to the APS-loaded groups. The greater recovery of MMP observed in the APS-loaded groups is consistent with previous reports that APS preserves MMP [25]. This observation suggests that extracellular free radical scavenging capability is not functionally equivalent to the reversal of intracellular oxidative damage. Under the hyperglycemic conditions characteristic of diabetes, a major factor driving the oxidative injury cascade is the continuous ROS leakage from the mitochondrial electron transport chain, rendering strategies that depend exclusively on extracellular scavengers unlikely to be sufficient to interrupt this endogenous driving force [58]. Consistent with the immunofluorescence findings, Western blotting showed coordinated upregulation of Nrf2 and its downstream antioxidant enzyme HO-1 in the APS-loaded groups, supporting an enhancement of the Nrf2/HO-1-associated intracellular antioxidant response. This response may also involve other Nrf2-regulated enzymes, including NQO1, while PINK1/Parkin-mediated mitophagy may contribute to the clearance of damaged mitochondria; however, these additional mechanisms were not directly examined in the present study [47]. These findings also highlight the importance of incorporating intracellular redox regulation into biomaterials for diabetic wounds, because extracellular antioxidant capacity alone may be insufficient to fully restore cellular function.

M2 polarization of macrophages is closely associated with enhanced mitochondrial OXPHOS, rather than being driven solely by anti-inflammatory signaling [59]. Against the early antibacterial and antioxidant background established by shell-layer TA, the sustained release of APS was associated with alleviated MMP depolarization and improved cellular bioenergetic profiles of macrophages, which may provide a favorable metabolic context for M2 polarization. Together with the increased expression of reparative macrophage-associated genes Arg-1, Ym1, and TGF-β, these findings support an association between mitochondrial bioenergetic recovery and macrophage phenotypic remodeling. The reduced oxidative burden associated with the shell-layer TA may attenuate the sustained mitochondrial oxidative stress, and TA and APS may thus exert complementary effects at the metabolic level. It is worth noting that TA, as a polyphenolic compound, has itself been reported to activate Nrf2, suggesting that the interaction between TA and APS may extend beyond their temporal division of function to encompass potential pathway-level cooperation, a hypothesis that warrants further validation through gene silencing or pathway blockade experiments [28]. Although this putative cooperative effect did not further enhance MVD or MPI, it may account for the lower collagen I/III ratio and improved collagen organization observed in AT@PCS.

Although the present study demonstrates the multifunctional effects of AT@PCS coaxial nanofibrous membranes in diabetic wound repair, several limitations should be acknowledged. With respect to material geometry, the planar fibrous architecture of AT@PCS imposes constraints on physical conformability to deep cavity wounds or geometrically irregular tissue defects, and future efforts may therefore explore the extension of this design to three-dimensional porous scaffolds or injectable fibrous systems to broaden clinical applicability [60]. In terms of antibacterial evaluation, although *E. coli* and *S. aureus* provide representative Gram-negative and Gram-positive bacterial models for initial assessment, diabetic infected wounds frequently involve more clinically challenging pathogens, such as methicillin-resistant *Staphylococcus aureus*, *Pseudomonas aeruginosa*, and polymicrobial biofilms. In addition, the present biofilm assay mainly supports inhibition of biofilm formation rather than disruption of established biofilms. Therefore, future studies should further evaluate the antibacterial efficacy of AT@PCS against clinically relevant drug-resistant bacteria, as well as its effects on biofilm formation and mature polymicrobial biofilms under more complex infection conditions. From a mechanistic perspective, although JC-1 staining, ATP production, OCR/ECAR analysis, and Nrf2/HO-1 protein expression collectively support improved mitochondrial bioenergetic function and activation of endogenous antioxidant defense, the causal dependence of APS-mediated mitochondrial protection on the Nrf2 pathway remains to be fully established. Future studies using Nrf2 inhibition or gene knockdown, together with direct mitochondrial ROS measurements, analysis of broader downstream antioxidant targets, and mitochondrial quality-control analyses, including mitochondrial fusion/fission dynamics, would help further clarify this mechanism. Concerning vascular assessment, blood perfusion in the wound bed was not directly assessed, and the relationship between structural vascular maturation and functional perfusion remains to be established. Regarding clinical translation, the present study addressed the susceptibility to aqueous dissolution of polysaccharide-based fibrous membranes through physical crosslinking with TA, achieving a hydrophilic yet water-insoluble material system. However, the long-term storage stability and scalable fabrication processes of the resulting dressing system remain to be optimized. Long-term wound recurrence and scar durability were not assessed within the current observation window and should be examined in future studies. Nevertheless, the present study provides experimental evidence that a temporally coordinated core-shell polysaccharide dressing can integrate antibacterial activity, antioxidant defense, and immunomodulation, offering a potential design strategy for future development of environmentally friendly wound dressings.

## 5 Conclusion

This study developed AT@PCS, a core-shell nanofibrous dressing fabricated from natural polysaccharides using a green, non-toxic solvent system. *In vitro* and *in vivo* evaluations demonstrated that this core-shell structure exhibits skin-mimicking mechanical compliance and favorable wet-state stability, supporting a temporally coordinated "outer defense and inner regulation” therapeutic strategy. The TA/QCS-modified shell rapidly reduced bacterial burden and scavenged extracellular ROS, establishing a low-oxidative-stress wound microenvironment. The core enabled sustained release of APS, which enhanced Nrf2/HO-1-associated antioxidant defense, improved mitochondrial bioenergetic function, and promoted macrophage polarization toward an M2-associated reparative phenotype, collectively facilitating wound healing. This study further indicates a functional distinction between extracellular antioxidant activity and intracellular mitochondrial energy metabolism regulation. Extracellular ROS scavenging alone may not be sufficient to alleviate the persistent mitochondrial oxidative stress observed in diabetic wounds. Overall, this fully natural core-shell system offers a promising strategy for designing multifunctional wound dressings based on temporally coordinated microenvironment regulation. Future studies should further elucidate the causal mechanisms underlying APS-mediated mitochondrial protection and immune regulation and extend this core-shell architecture to three-dimensional porous scaffolds or injectable formulations to meet the clinical demands of geometrically complex wounds.

## CRediT authorship contribution statement

**Yanqi Chen:** Writing – original draft, Methodology, Investigation, Data curation. **Huihui Zhang:** Writing – review & editing, Methodology, Investigation. **Tingzi Zhao:** Investigation, Data curation, Conceptualization. **Hai Zhou:** Funding acquisition, Conceptualization. **Zhi Xu:** Visualization, Supervision, Methodology. **Jiaqi Liang:** Software, Investigation, Data curation. **Yixiang HePeng:** Formal analysis, Data curation. **Chaoyang Huang:** Resources, Methodology, Funding acquisition. **Xu Wu:** Supervision, Funding acquisition. **Lianglong Chen:** Validation, Supervision, Methodology, Funding acquisition. **Lei Yang:** Supervision, Project administration, Methodology, Funding acquisition.

## Funding

This work was financially supported by the Marine Economy Development Special Fund (Six Marine Industries) under the 10.13039/100020730Department of Natural Resources of Guangdong Province (GDNRC [2024]27).

## Declaration of competing interest

We declare that no conflict of interest exits in the submission of this manuscript, and manuscript is approved by all authors for publication. I would like to declare on behalf of my co-authors that the work described was original research that has not been published previously, and not under consideration for publication elsewhere, in whole or in part. All the authors listed have approved the manuscript that is enclosed.

## Data Availability

Data will be made available on request.

## References

[1] Xiong Y., Knoedler S., Alfertshofer M., Kim B.S., Jiang D., Liu G., Rinkevich Y., Mi B. (2025). Mechanisms and therapeutic opportunities in metabolic aberrations of diabetic wounds: a narrative review. Cell Death Dis..

[2] Palma F.R., Gantner B.N., Sakiyama M.J., Kayzuka C., Shukla S., Lacchini R., Cunniff B., Bonini M.G. (2024). ROS production by mitochondria: function or dysfunction?. Oncogene.

[3] Zhao L., Zhang C., Li J., Guo Y., Meng J., Yin Y., Liu W., Kang J., Jiang Y., Ning B. (2026). Dynamic ROS-responsive injectable hydrogel incorporating nanozymes for spinal cord repair by alleviating oxidative stress and neuronal ferroptosis. Biomaterials.

[4] Xu X., Pang Y., Fan X. (2025). Mitochondria in oxidative stress, inflammation and aging: from mechanisms to therapeutic advances. Signal Transduct. Targeted Ther..

[5] Wu K.K., Xu X., Wu M., Li X., Hoque M., Li G.H.Y., Lian Q., Long K., Zhou T., Piao H., Xu A., Hui H.X., Cheng K.K. (2024). MDM2 induces pro-inflammatory and glycolytic responses in M1 macrophages by integrating iNOS-nitric oxide and HIF-1alpha pathways in mice. Nat. Commun..

[6] Chen S., Saeed A., Liu Q., Jiang Q., Xu H., Xiao G.G., Rao L., Duo Y. (2023). Macrophages in immunoregulation and therapeutics. Signal Transduct. Targeted Ther..

[7] Wang Y., Chang F., Li Y., Wang F., Li C., Li H., Jiang Y. (2025). Bi(2)WO(6)@Cu(2)O-GO(x) bio-heterojunction(p-n) spray for accelerating chronic diabetic wound repairment with bilaterally enhanced sono-catalysis and glycolytic inhibition antisepsis. Biomaterials.

[8] Xie Y., Yu Y., Cai Z., Sun J., Bi W., Yang F., Zhou Q., Wu X., Li R., Zhu K., Yu Y., Cui W., Liu W., Sun Y. (2025). The injectable cationic double-crosslinked hydrogel facilitates bone regeneration and repair through sustained antibacterial activity. Mater. Today Bio.

[9] Jin C., Chen J., Miao Y., Tian X., Wang J., He F., Liu Y., Xu Y. (2025). Cuttlefish ink nanoparticle-engineered hydrogel microspheres synergistically attenuate disc degeneration via antioxidant defense and matrix synthesis activation. Mater. Today Bio.

[10] Chen L., Zhan M., Zhang L., Xiong Z., Zhang C., Zhang X., Yang Z., Ma L., Cheng Y., Chen H. (2025). Dual-layer microneedles for immunomodulation and infection-responsive therapy in diabetic foot ulcers. Mater. Today Bio.

[11] Guo J., Hu B., Wei Y., Cheng G., Wang C., Qin X., Chen X., Chen J., Chen Z., Chen T. (2025). Hydrogel delivering self-assembled herbal nanoparticles accelerates diabetic wound healing through mitochondrial regulation. Mater. Today Bio.

[12] Zhou X., Huang Z., Yang H., Jin L., Zhang Y. (2025). Mitochondrial-targeting drug-loaded nanoparticles reprogram macrophage metabolism via ROS/NO Co-elimination for diabetic wound healing. ACS Appl. Mater. Interfaces.

[13] Huang X., Lin Z., Ruan M., Huang P., Ding H., Pan H., Cao J., Ma C., Zhao Q., Guo W., Wu K., Fang C., Liu A., Zheng L. (2025). Light up the mitochondria: smart tungstate-oligosaccharide nanoplatform orchestrates mitochondrial transfer from M2 macrophages to restore endothelial function for adaptive diabetic wound regeneration. Mater. Today Bio.

[14] Cui M., He H., Lu H., Yang X., Chen S., Zhou X., Li F., He C., Yang G. (2026). Skin-mimetic bilayer hydrogel normalizes diabetic wound healing by orchestrating inflammatory cell dynamics: an early intervention strategy. Bioact. Mater..

[15] Bian H., Song F., Wang S., Sun W., Hu B., Liang X., Yang H., Huang C. (2025). Matrix vesicle-inspired delivery system based on nanofibrous chitosan microspheres for enhanced bone regeneration. Mater. Today Bio.

[16] Priya S., Choudhari M., Tomar Y., Desai V.M., Innani S., Dubey S.K., Singhvi G. (2024). Exploring polysaccharide-based bio-adhesive topical film as a potential platform for wound dressing application: a review. Carbohydr. Polym..

[17] Qu Z., Wang Y., Dong Y., Li X., Hao L., Sun L., Zhou L., Jiang R., Liu W. (2024). Intelligent electrospinning nanofibrous membranes for monitoring and promotion of the wound healing. Mater. Today Bio.

[18] Yao H., Wu M., Lin L., Wu Z., Bae M., Park S., Wang S., Zhang W., Gao J., Wang D., Piao Y. (2022). Design strategies for adhesive hydrogels with natural antibacterial agents as wound dressings: status and trends. Mater. Today Bio.

[19] Huang C., Chen L., Zhang H., Liu B., Zhou H., Chen Y., Li X., Liu X., Zhao L., Wang X., Wu M., Li S., Yi D., Liu C., Pan H., Yang L. (2025). Feather-inspired janus interfaces with spatiotemporal ionic programming for diabetic wound infection control and regenerative healing. Mater. Today Bio.

[20] Gao W., Liu Y., Wu S. (2025). Properties of pullulan and its effects on starch gelatinization, retrogradation, and protein interaction: a review. Food Chem..

[21] Tian Y., Cui Y., Ren G., Fan Y., Dou M., Li S., Wang G., Wang Y., Peng C., Wu D. (2024). Dual-functional thermosensitive hydrogel for reducing infection and enhancing bone regeneration in infected bone defects. Mater. Today Bio.

[22] Luo H., Xu H., Zhang H., Li X., Wu Q., Gao T. (2025). Photodynamic therapy combined with quaternized chitosan antibacterial strategy for instant and prolonged bacterial infection treatment. Carbohydr. Polym..

[23] Chen T., Xie L., Shen M., Yu Q., Chen Y., Xie j. (2024). Recent advances in astragalus polysaccharides: structural characterization, bioactivities and gut microbiota modulation effects. Trends Food Sci. Technol..

[24] Li R., Chen W.-c., Wang W.-p., Tian W.-y., Zhang X.-g. (2010). Antioxidant activity of astragalus polysaccharides and antitumour activity of the polysaccharides and siRNA. Carbohydr. Polym..

[25] Liu X., Guo C., Yang W., Wang W., Diao N., Cao M., Cao Y., Wang X., Wang X., Pei H., Jiang Y., Kong M., Chen D. (2024). Composite microneedles loaded with Astragalus membranaceus polysaccharide nanoparticles promote wound healing by curbing the ROS/NF-kappaB pathway to regulate macrophage polarization. Carbohydr. Polym..

[26] Sun J., Wang X., Wang X., Yu W., Yu Y., Ge S., Dong Z. (2026). Tannic acid-assisted mechanical training transforms natural hydrogels into robust and bioactive membranes for guided bone regeneration. Mater. Today Bio.

[27] Chen H., Luo Z., Chen Q., Chang Y., Zheng J. (2026). Physical crosslinking in double network hydrogels: structural design, toughening mechanisms, and functional diversity. Prog. Polym. Sci..

[28] Hosseini M., Moghaddam L., Barner L., Cometta S., Hutmacher D.W., Medeiros Savi F. (2025). The multifaceted role of tannic acid: from its extraction and structure to antibacterial properties and applications. Prog. Polym. Sci..

[29] Yu B., Zhou D., Wang F., Chen X., Li M., Su J. (2025). Organoids for tissue repair and regeneration. Mater. Today Bio.

[30] Li Z., Guo Q., Chen R., E Y., Wang Y., Zhu M., Shi G., Hao Z., Li J., Zhu S. (2025). Tannic acid coated core-shell fibers with antibacterial and antioxidant properties for diabetic wound healing. Mater. Des..

[31] Li Y., Wang X., Miao C., Song M., Cao Z. (2025). Quaternized chitosan/salvianolic acid B multifunctional hydrogel with ROS/glucose dual responsive properties for diabetic wound healing. Carbohydr. Polym..

[32] Pan W., Qi X., Xiang Y., You S., Cai E., Gao T., Tong X., Hu R., Shen J., Deng H. (2022). Facile formation of injectable quaternized chitosan/tannic acid hydrogels with antibacterial and ROS scavenging capabilities for diabetic wound healing. Int. J. Biol. Macromol..

[33] Qiu Y.L., Li Y., Zhang G.L., Hao H., Hou H.M., Bi J. (2024). Quaternary-ammonium chitosan, a promising packaging material in the food industry. Carbohydr. Polym..

[34] Pissarenko A., Meyers M.A. (2020). The materials science of skin: analysis, characterization, and modeling. Prog. Mater. Sci..

[35] Han X., Gao W., Li L., Gao Z., Wang C. (2025). Charge gating-enhanced electrostatic targeting using quaternary-ammonium chitosan@metal organic framework for rapid bacterial detection and synergistic antibacterial therapy. Int. J. Biol. Macromol..

[36] Cheng S., Wang H., Pan X., Zhang C., Zhang K., Chen Z., Dong W., Xie A., Qi X. (2022). Dendritic hydrogels with robust inherent antibacterial properties for promoting bacteria-infected wound healing. ACS Appl. Mater. Interfaces.

[37] Yeow J., Luo M., Chng S.S. (2023). Molecular mechanism of phospholipid transport at the bacterial outer membrane interface. Nat. Commun..

[38] Zheng Y., Sun J., Luo Z., Li Y., Huang Y. (2024). Emerging mechanisms of lipid peroxidation in regulated cell death and its physiological implications. Cell Death Dis..

[39] Jiang T., Liu S., Wu Z., Li Q., Ren S., Chen J., Xu X., Wang C., Lu C., Yang X., Chen Z. (2022). ADSC-exo@MMP-PEG smart hydrogel promotes diabetic wound healing by optimizing cellular functions and relieving oxidative stress. Mater. Today Bio.

[40] Tong J., Sun D., Sun J., Zhang J., Chen M., Liu X., Zhang R., Zhang F., Zheng C., Wei Y., Guo X. (2025). Intelligent responsive PVA hydrogel loaded with composite MXene nanozyme enables efficient myocardial infarction repair by disrupting the oxidative stress-inflammation cascade. Mater. Today Bio.

[41] Ge W., Qi L., Wang Y., Wang J., Fang X., Lei S., Lin D., Zhang L., Zhang S. (2026). Engineered M2 macrophage-derived extracellular vesicles reprogram mitochondrial metabolism to alleviate temporomandibular joint cartilage degeneration. Mater. Today Bio.

[42] Qi X., Li Y., Xiang Y., Chen Y., Shi Y., Ge X., Zeng B., Shen J. (2025). Hyperthermia-enhanced immunoregulation hydrogel for oxygenation and ROS neutralization in diabetic foot ulcers. Cell Biomaterials.

[43] Cui Y., Jiang X., Yang M., Yuan Y., Zhou Z., Gao X., Jia G., Cao L., Li D., Zhao Y., Zhang X., Zhao G. (2023). SEMA4D/VEGF surface enhances endothelialization by diminished-glycolysis-mediated M2-like macrophage polarization. Mater. Today Bio.

[44] Wang X., Li R., Zhao H. (2024). Enhancing angiogenesis: innovative drug delivery systems to facilitate diabetic wound healing. Biomed. Pharmacother..

[45] He Y., Sheng J., Liu F., Li F., Lu S., Chen W., Yang L., Zhou P., Chen Z., Chen S., Luo Z., Sun J. (2025). SHED-derived exosome-mimetics promotes rotator cuff tendon-bone healing via macrophage immunomodulation through NF-kappaB suppression and autophagy activation. Mater. Today Bio.

[46] Zhang Q., Wang T., Ren J., Guo Y., Yin Z., Zhang C., Fan H., Ren Y., Da J., Hu N. (2025). Multifunctional astragalus polysaccharide composited microneedle promotes oral ulcers healing by inhibiting ferroptosis via Nrf2/HO-1/SLC7A11 axis. Mater. Today Bio.

[47] Yang X., Liu Y., Cao J., Wu C., Tang L., Bian W., Chen Y., Yu L., Wu Y., Li S., Shen Y., Xia J., Du J. (2025). Targeting epigenetic and post-translational modifications of NRF2: key regulatory factors in disease treatment. Cell Death Dis..

[48] Li Y., Wang Y., Wang Y., Wang F., Chang F., Jiang Y. (2025). Defect-Rich MoO(3-X)@CuO(2) nanosheets mediated ultrasound-enhanced cuproptosis antibacterial activity and M2 macrophage reprogramming for optimizing diabetic wound repairment. Adv. Healthcare Mater..

[49] Li H., Liu H., Chen R., Zheng S., Xu Y., Lei M., Mai H., Wang H., Cao T., Li L., Zhang Y., Wang J., Cheng H. (2026). Electrospun dressing promotes diabetic-infected wound healing via rapid and sustained antibacterial, ROS scavenging, and immune modulation ability. J. Nanobiotechnol..

[50] Xia H., Tang Q., Chen Z., Cao S., Wu L., Hao L., Hu X., Sun L., Gu Z., Mao H. (2026). Engineered polypeptide cascade-release platform restores macrophage plasticity for accelerated diabetic wound healing. Bioact. Mater..

[51] Wang H., Wang L., Liu Z., Luo Y., Kang Z., Che X. (2024). Astragaloside/PVP/PLA nanofiber functional dressing prepared by coaxial electrostatic spinning technology for promoting diabetic wound healing. Eur. Polym. J..

[52] Xu M., Yao X., Wang Y., Liu H., Yang W., Zhang P. (2025). Electrospun miRNA-21@doxycycline core-sheath nanofibers for a synergistic treatment of diabetic wounds. Compos. Commun..

[53] Tang L., Xie S., Wang D., Wei Y., Ji X., Wang Y., Zhao N., Mou Z., Li B., Sun W.R., Wang P.Y., Basmadji N.P., Pedraz J.L., Vairo C., Lafuente E.G., Ramalingam M., Xiao X., Wang R. (2025). Astragalus polysaccharide/carboxymethyl chitosan/sodium alginate based electroconductive hydrogels for diabetic wound healing and muscle function assessment. Carbohydr. Polym..

[54] Wang T.Y., Zhu X.Y., Wu F.G. (2023). Antibacterial gas therapy: strategies, advances, and prospects. Bioact. Mater..

[55] Yan W., Shi M., Dong C., Liu L., Gao C. (2020). Applications of tannic acid in membrane technologies: a review. Adv. Colloid Interface Sci..

[56] Guo Q., Tang Y., Wang S., Xia X. (2025). Applications and enhancement strategies of ROS-based non-invasive therapies in cancer treatment. Redox Biol..

[57] Xiang Y., Pan Z., Qi X., Ge X., Xiang J., Xu H., Cai E., Lan Y., Chen X., Li Y., Shi Y., Shen J., Liu J. (2024). A cuttlefish ink nanoparticle-reinforced biopolymer hydrogel with robust adhesive and immunomodulatory features for treating oral ulcers in diabetes. Bioact. Mater..

[58] Giri B., Dey S., Das T., Sarkar M., Banerjee J., Dash S.K. (2018). Chronic hyperglycemia mediated physiological alteration and metabolic distortion leads to organ dysfunction, infection, cancer progression and other pathophysiological consequences: an update on glucose toxicity. Biomed. Pharmacother..

[59] Li J., Li Z., Han L., Xu N., Jia J., Hu A., Chen Y., Liu C., Zhang X., Zhu W., Yu Y., Luo G. (2026). Macrophage metabolic reprogramming by vanadium released from glucose-responsive bio-gel accelerates diabetic wound repair. Signal Transduct. Targeted Ther..

[60] Xue Y.T., Chen M.Y., Cao J.S., Wang L., Hu J.H., Li S.Y., Shen J.L., Li X.G., Zhang K.H., Hao S.Q., Juengpanich S., Cheng S.B., Wong T.W., Yang X.X., Li T.F., Cai X.J., Yang W. (2023). Adhesive cryogel particles for bridging confined and irregular tissue defects. Milit. Med. Res..

