# Supplementary material for: A biomimetic core-shell nanofibrous dressing for temporally coordinated infection control and mitochondrial protection in diabetic wounds
Source: Mater Today Bio. 2026 Aug 28;40:103599. doi: 10.1016/j.mtbio.2026.103599 (PMC13560321; doi:10.1016/j.mtbio.2026.103599)
Supplement: Supplementary file 1 [file mmc1.docx]

**Supporting Information**

A biomimetic core-shell nanofibrous dressing for temporally coordinated infection control and mitochondrial protection in diabetic wounds

Yanqi Chen^1†^, Huihui Zhang^1†^, Tingzi Zhao^1†^, Hai Zhou^1^, Zhi Xu^3^, Jiaqi Liang^1^, Yixiang HePeng^2^, Chaoyang Huang^1*^, Xu Wu^2*^, Lianglong Chen^1,4*^, Lei Yang^1*^

**Affiliations**

^1^Department of Burns, Nanfang Hospital, Southern Medical University, Guangzhou, 510515, China.

^2^Department of Thoracic Surgery, Nanfang Hospital, Southern Medical University, Guangzhou, 510515, China.

^3^Huiqiao Medical Center, Nanfang Hospital, Southern Medical University, Guangzhou, 510515, China.

^4^Department of Plastic Surgery, Huizhou Central People's Hospital, No. 41 Eling North Road, Huicheng District, Huizhou City, Guangdong Province, 516001, China

†These authors have contributed equally to this work.

*Corresponding authors: Chaoyang Huang, E-mail: 1194593484@qq.com; Xu Wu, E-mail: wuxu_southhospital@163.com; Lianglong Chen, E-mail: cllwql@163.com; Lei Yang, E-mail: yuanyang@smu.edu.cn.

**Fig. S1. (A)** Macroscopic morphology of the four dressings in their dry states, namely PCS, A@PCS, T@PCS, and AT@PCS. **(B)** Assessment of water-resistant stability following hydration of each sample group. Upon exposure to aqueous conditions, uncrosslinked PCS and A@PCS underwent pronounced swelling and gelation, accompanied by structural collapse, whereas T@PCS and AT@PCS, reinforced through a polyphenol-polysaccharide physical crosslinking network, demonstrated effective resistance to swelling and retained structural integrity under aqueous conditions. **(C)** Transmission electron microscopy image of an individual nanofiber constituent of AT@PCS. **(D)** Representative fluorescence microscopy images of an individual AT@PCS core-shell fiber. Rhodamine B (red) and calcein (green) were used to label the core and shell layers, respectively. **(E)** Force-displacement curves of water-wetted T@PCS and AT@PCS nanofibrous membranes. **(F)** Sequential images of the dynamic adhesion procedure of the AT@PCS dressing applied to the finger joint (Steps 1-5), wherein the dressing conformed adaptively to joint flexion without detachment, thereby substantiating its conformability and adhesion and self-adhesive properties.

**Fig. S2.** Korsmeyer-Peppas fitting of APS release from AT@PCS under pH 5.4 and pH 7.4 conditions. The release exponent n and R^2^ were obtained by linear regression of ln(Mt/M∞) versus ln(t).

**Fig. S3.** **(A)** Moisture retention rate of the different nanofibrous membranes. **(B)** Fluid absorption ratio of T@PCS and AT@PCS after 1 h.

**Fig. S4.** Inhibitory effects of various samples on *S. aureus* biofilm formation.

**Fig. S5. (A)** DPPH radical-scavenging activity and **(B)** cumulative TA release profiles of T@PCS and AT@PCS over time.

**Fig. S6. (A)** Representative images of the DPPH scavenging assay. **(B)** Representative JC-1 fluorescence micrographs of RAW 264.7 macrophages cultured under oxidative stress and treated with the corresponding material extracts (scale bar = 50 µm). **(C)** Quantification of JC-1 aggregate (red) fluorescence intensity in the images shown in **(B)**. **(D)** Intracellular ATP content in RAW 264.7 macrophages from each group. **(E)** Seahorse OCR traces of RAW 264.7 macrophages under different treatments. **(F)** Quantification of basal respiration derived from OCR analysis. **(G)** Quantification of maximal respiration derived from OCR analysis. **(H)** Seahorse ECAR traces of RAW 264.7 macrophages under different treatments. **(I)** Quantification of glycolysis derived from ECAR analysis. **(J)** Quantification of maximal glycolytic capacity derived from ECAR analysis.

**Fig. S7.** Quantification of Nrf2 and HO-1 protein expression by Western blotting.

**Fig. S8.** **(A)** Immunofluorescence staining images of IL-6 and CD163 (scale bar = 100 µm). **(B)** Quantitative analysis of IL-6 fluorescence intensity. **(C)** Quantitative analysis of CD163 fluorescence intensity. **(D-G)** Relative mRNA levels of Arg-1, Ym1, TGF-β, and IL-1β determined by qPCR. **(H)** Flow cytometric quantification of the proportion of CD86^+^ (M1 phenotype) cells among RAW 264.7 macrophages. **(I)** Quantitative analysis by flow cytometry of the proportion of CD206^+^ (M2 phenotype) cells among RAW 264.7 macrophages.

**Fig. S9. (A)** Representative VEGF immunofluorescence staining images (scale bar = 100 µm) and representative tube-formation images of HUVECs (scale bar = 200 µm) cultured with conditioned media (CM) from differently treated RAW 264.7 macrophages. **(B)** Quantitative analysis of VEGF fluorescence intensity. **(C,D)** Quantitative analysis of junction and mesh numbers in the tube-formation assay.

**Fig. S10. (A)** Representative photographs of wounds 2 days after bacterial inoculation (treatment day 0), prior to dressing application. **(B)** H&E staining of wound tissue on day 3 post-treatment (scale bars = 2 mm for upper panels, 150 µm for lower panels). **(C)** Histological scoring of inflammatory cell infiltration in H&E-stained wound sections on day 3 post-treatment. **(D)** Representative photographs and cumulative *in vivo* degradation of AT@PCS nanofibrous membranes over 4 days. **(E)** Evaluation of epidermal thickness and collagen deposition in wound tissues on treatment day 7.

**Fig. S11.** Quantification of **(A)** microvessel density (MVD) and **(B)** microvessel pericyte coverage index (MPI) in wound tissues collected on treatment day 7.

**Table S1.** Drug loading and encapsulation efficiency of APS

| Group | Batch | Theoretical APS Input (mg/membrane) | Measured APS (mg/membrane) | LC (%) | EE (%) |
| --- | --- | --- | --- | --- | --- |
| A@PCS | 1 | 18.18 | 16.566 | 16.566 | 91.12 |
|  | 2 |  | 16.834 | 16.834 | 92.59 |
|  | 3 |  | 16.029 | 16.029 | 88.17 |
|  | Mean ± SD |  | 16.476 ± 0.410 | 16.476 ± 0.410 | 90.63 ± 2.25 |
|  | RSD (%) |  | 2.49 | 2.49 | 2.48 |
| AT@PCS | 1 | 8.42 | 7.438 | 7.438 | 88.34 |
|  | 2 |  | 7.216 | 7.216 | 85.70 |
|  | 3 |  | 7.623 | 7.623 | 90.53 |
|  | Mean ± SD |  | 7.426 ± 0.204 | 7.426 ± 0.204 | 88.19 ± 2.42 |
|  | RSD (%) | 0 | 2.74 | 2.74 | 2.74 |

Data are presented as mean ± SD of three independent batches. LC, drug loading; EE, encapsulation efficiency; RSD, relative standard deviation across batches (inter-batch reproducibility). Experimental details are provided in the Methods.

**Supplementary Experimental Methods**

1. To visualize the core-shell architecture, rhodamine B and calcein were added to the pre-mixed core and shell spinning solutions at final concentrations of 5 and 2 mg/mL, respectively. After dye addition, both solutions were stirred for 30 min in the dark and subsequently subjected to coaxial electrospinning under light-protected conditions. The resulting fibers were examined using an inverted fluorescence microscope (Nikon, N31373), and their core-shell architecture was characterized based on the spatial distribution of the two fluorescent dyes.

2. Membranes from each group were cut into rectangular specimens (1 cm × 6 cm), and their tensile properties were measured using a universal testing machine. To evaluate their mechanical behavior after localized wetting, 5 μL of deionized water was applied to the center of the T@PCS and AT@PCS specimens, followed by tensile testing using the same parameters as those applied under dry conditions. Because the PCS and A@PCS membranes rapidly disintegrated upon contact with water and could not maintain sufficient structural integrity for clamping, their tensile properties under wetted conditions could not be determined.

3. Released APS was quantified by high-performance liquid chromatography coupled with an evaporative light-scattering detector (HPLC-ELSD). Separation was performed on a Waters Symmetry C18 column (250 mm × 4.6 mm, 5 μm) maintained at 30 ℃. The mobile phase consisted of 0.02% aqueous formic acid and acetonitrile. The proportion of acetonitrile was increased from 16% to 49% over 0–30 min and subsequently returned to 16% over 30–50 min. The flow rate was 1.0 mL/min, and the injection volume was 40 μL. The ELSD was operated at a gas pressure of 275.8 kPa (40 psi) and a drift-tube temperature of 65 °C. APS concentrations were determined from the corresponding peak areas using a calibration curve constructed with APS standards subjected to the same sample-preparation procedure.

4. APS loading and encapsulation efficiency. The actual APS loading was determined prior to release profiling by complete extraction followed by HPLC quantification. Accurately weighed membranes were fully dissolved and sonicated to release the encapsulated APS, and the extract was quantified by HPLC. Drug loading (LC) and encapsulation efficiency (EE) were calculated as LC (%) = (measured APS mass / membrane mass) × 100 and EE (%) = (measured APS mass / theoretical APS input) × 100. Measurements were performed on three independent batches, and the experimentally determined loading was used as ML in the cumulative-release calculation. The inter-batch RSD was below 5% for all groups (**Table S1**).

5. Moisture retention test. Glycerin (Gl) was used as a positive control. Samples were dried to a constant weight (m_0_), exposed for 30 min to an atmosphere of 81% relative humidity maintained using a saturated ammonium sulfate solution, and immediately weighed (m_1_). The samples were subsequently transferred to a dry desiccator pre-equilibrated for 72 h and weighed again after 1 h (m_2_). The moisture retention rate was calculated as Rh (%) = [(m_2_ − m_0_)/(m_1_ − m_0_)] × 100, where m_0_, m_1_, and m_2_ represent the initial dry weight, the weight after moisture absorption, and the weight after subsequent desiccation, respectively.

6. PBS absorption. Dressings of uniform thickness were cut into equal-sized specimens and weighed (W_0_). After immersion in PBS (pH 7.4) at 37 ℃ for 1 h, the specimens were gently blotted to remove excess surface liquid and immediately weighed (W_c_). PBS absorption was calculated as follows: PBS absorption (%) = [(W_c_ − W_0_)/W_0_] × 100, where W_0_ and W_c_ represent the initial dry weight and the weight after PBS absorption, respectively.

7. *In vitro* release of TA. The release of TA from T@PCS and AT@PCS nanofibrous membranes was evaluated in phosphate-buffered saline (PBS, pH 7.4) at 37 ℃. Equal-sized membrane specimens were separately placed in dialysis bags containing 2 mL of PBS, and the sealed bags were immersed in Erlenmeyer flasks containing 98 mL of PBS (pH 7.4). The flasks were incubated in a shaking water bath at 37 ℃. At predetermined time points, the amount of TA released into the medium was quantified using a UV-Vis spectrophotometer at 275 nm based on a TA calibration curve prepared in PBS (pH 7.4). The cumulative TA release was normalized to the initial membrane area.

8. Mitochondrial Function and Cellular Energy Metabolism Assays. Intracellular ATP levels were measured using a luciferase-based ATP assay kit according to the manufacturer’s instructions. Cells from each group were lysed with ATP lysis buffer, and the lysates were centrifuged to collect the supernatants. The ATP working solution was then added to the supernatants, and luminescence intensity was detected using a luminometer. ATP content was calculated according to the standard curve and normalized to the total protein concentration of each sample.

Cellular bioenergetic profiles were examined with a Seahorse XFe96 extracellular flux analyzer. RAW 264.7 macrophages were plated in Seahorse XF microplates at 2 × 10^4^ cells per well and cultured overnight. Prior to the assay, the medium was exchanged for Seahorse XF assay medium, followed by equilibration for 45-60 min at 37 ℃ in a CO_2_-free incubator. Mitochondrial respiration was evaluated by monitoring OCR during a standard mitochondrial stress test. After baseline measurement, oligomycin, FCCP, and rotenone/antimycin A were sequentially injected to generate the mitochondrial stress profile. Basal and maximal respiration were calculated from the OCR traces using Seahorse Wave software after subtracting non-mitochondrial respiration. Glycolytic metabolism was analyzed by recording ECAR during a glycolysis stress test. ECAR was measured at baseline and after sequential injection of glucose, oligomycin, and 2-deoxy-D-glucose, from which glycolysis and glycolytic capacity were calculated using Seahorse Wave software. OCR and ECAR values were normalized to cell number and expressed as pmol/min and mpH/min, respectively.

9. Total RNA was extracted from RAW 264.7 macrophages using TRIzol reagent according to the manufacturer’s instructions. The concentration and purity of RNA were determined using a spectrophotometer, and cDNA was synthesized using a reverse transcription kit. Quantitative real-time PCR was performed using SYBR Green master mix on a real-time PCR system. The relative mRNA expression levels of IL-1β, Arg-1, Ym1, and TGF-β were normalized to GAPDH and calculated using the 2^-ΔΔCt^ method.

10. To evaluate the paracrine effects of treated macrophages on endothelial cells, CM were collected from RAW 264.7 macrophages in different groups, including untreated control, HG/LPS-stimulated model, and HG/LPS-stimulated macrophages treated with extracts from PCS, A@PCS, T@PCS, or AT@PCS. After the designated treatments, the macrophages were washed three times with PBS to remove residual treatment components and subsequently incubated in an equivalent volume of serum-free DMEM for an additional 24 h. The culture supernatants were collected and centrifuged at 1000 × g for 10 min at 4 ℃ to remove cells and cellular debris. The clarified supernatants were mixed with fresh endothelial growth medium at a ratio of 1:1 to prepare CM. HUVECs were then cultured with the prepared CM for 24 h before VEGF immunofluorescence staining or seeded onto polymerized Matrigel and cultured with CM for 12 h for the tube formation assay, following the procedures described above.

11. Histological scoring of inflammatory cell infiltration. Inflammatory cell infiltration was semiquantitatively evaluated in H&E-stained wound sections using a previously reported scoring system [1]. The infiltration of polymorphonuclear and mononuclear cells was graded on a 0-3 scale as follows: 0, absence of inflammation; 1, discrete inflammation with few inflammatory cells; 2, moderate inflammation with numerous inflammatory cells; and 3, severe inflammation characterized by extensive inflammatory cell infiltration. The resulting scores were used for statistical analysis.

12. Cumulative *in vivo* degradation. AT@PCS nanofibrous membrane discs (1 cm in diameter) were dried to a constant weight, and their initial dry mass was recorded as m_0_. The membranes were subsequently implanted subcutaneously in the dorsal region of rats, and the incisions were closed. At predetermined time points, the membranes were retrieved, oven-dried to a constant weight, and weighed (m_t_). The cumulative degradation was calculated as follows: Cumulative degradation (%) = [(m_0_ − m_t_)/m_0_] × 100, where m_0_ is the initial dry mass and mₜ is the residual dry mass at time t.

13. Quantification of MVD and pericyte coverage. Merged CD31/α-SMA immunofluorescence images obtained from the same tissue sections were analyzed using ImageJ. A single CD31-positive endothelial cell or a discrete CD31-positive endothelial cell cluster, with or without a visible lumen, was counted as one microvessel. MVD was calculated as the number of CD31-positive microvessels divided by the analyzed tissue area and expressed as vessels mm^-2^. A CD31-positive microvessel associated with perivascular α-SMA-positive mural cell coverage was considered a mature, pericyte-covered microvessel. The MPI was calculated as the percentage of CD31-positive microvessels exhibiting α-SMA-positive mural cell coverage.

**References**

[1] L.R. de Moura Estevao, P. Cassini-Vieira, A.G.B. Leite, A.A.V. de Carvalho Bulhoes, L. da Silva Barcelos, J. Evencio-Neto, Morphological Evaluation of Wound Healing Events in the Excisional Wound Healing Model in Rats, Bio Protoc 9(13) (2019) e3285. https://doi.org/10.21769/BioProtoc.3285.
